# State-Dependent and Social Modulation of Circulating Glucocorticoids in a Nomadic Songbird, the Red Crossbill (*Loxia Curvirostra*)

**DOI:** 10.1093/iob/obaf047

**Published:** 2025-12-09

**Authors:** B J Vernasco, I T Moore, J M Cornelius, H E Watts

**Affiliations:** School of Biological Sciences, Washington State University, Pullman, WA 99164, USA; Current affiliation: Department of Biology, Whitman College, Walla Walla, WA 99362, USA; Department of Biological Sciences, Virginia Tech, Blacksburg, VA 24061, USA; Department of Integrative Biology, Oregon State University, Corvallis, OR 97331, USA; Current affiliation: Department of Biology, Whitman College, Walla Walla, WA 99362, USA; Center for Reproductive Biology, Washington State University, Pullman, WA 99164, USA

## Abstract

Glucocorticoids facilitate the integration of environmental information and coordination of organismal responses to perturbations. Circulating glucocorticoids are hypothesized to depend on an individual’s environment and condition (i.e., state) to facilitate surviving challenges while minimizing fitness costs. Studies specifically focused on sources of individual variation in circulating glucocorticoids are critical to understanding state-dependent modulation of glucocorticoids and integrated phenotypes more broadly. Such studies can also provide insight into the evolution and adaptive significance of circulating glucocorticoids. Here, we repeatedly sample individuals before and during food restriction to identify how and when food availability and intrinsic differences (i.e., body condition and telomere length), including those of social partners, covary with glucocorticoids in captive Red Crossbills (*Loxia curvirostra*), a nomadic songbird that specializes on foraging for conifer seeds. Conifer seeds are ephemeral resources produced during unpredictable, but locally synchronous, masting events. Fluctuating food availability and social cues, change the behavior and glucocorticoid physiology of Red Crossbills. Pairs consisting of an adult and juvenile were food restricted using an environmental manipulation known to induce socially mediated changes in glucocorticoid signaling. Baseline and stress-induced glucocorticoids were measured before and during food restriction. Amongst adults, stress-induced glucocorticoids declined following food restriction and positively covaried with telomere length, independent of food availability. These results support the hypothesis that the acute glucocorticoid response is adaptively modulated based on environmental conditions and individual differences in state as measured by telomere length. Under food restriction, juvenile baseline glucocorticoids negatively covaried with body condition and the telomere lengths of adult social partners. The covariation between adult telomere lengths and juvenile baseline glucocorticoids suggests that telomere lengths of adults may relate to adult phenotypes, a hypothesis supported by the covariation between adult telomeres and stress-induced glucocorticoids. Further, as patterns were absent before food restriction, our results demonstrate how environmental challenges can reveal the importance of intrinsic differences to organismal responses and social cues. This study leverages a non-model organism experiencing an ecologically relevant environmental challenge to exemplify how intrinsic differences, including those of social partners, can modulate an endocrine mediator of organismal responses to environmental perturbations.

## Introduction

Understanding how organisms respond to environmental challenges is a fundamental biological question that is dependent on integrative studies bridging different levels of biological organization (Ricklefs and Wikelski 2002; Chevin et al. 2010). Differences in the magnitude of the environmental challenge can contribute to variation in organismal responses, but individual variation in responses to the same environmental challenges is also a highly relevant, though relatively understudied, source of variability (Dall et al. 2012; Moiron et al. 2020). Two causes of variation in how individuals respond to environmental challenges are intrinsic differences, such as age or condition, and variability in an individual’s social environment (Bennett 1987; Webster and Ward 2011; Creel et al. 2013). Repeatedly sampling individuals under contrasting environmental conditions can provide valuable insight into how intrinsic factors and characteristics of the social environment mediate individual differences in responses to environmental challenges (Williams 2008; Malkoc et al. 2022).

**Fig. 1:**
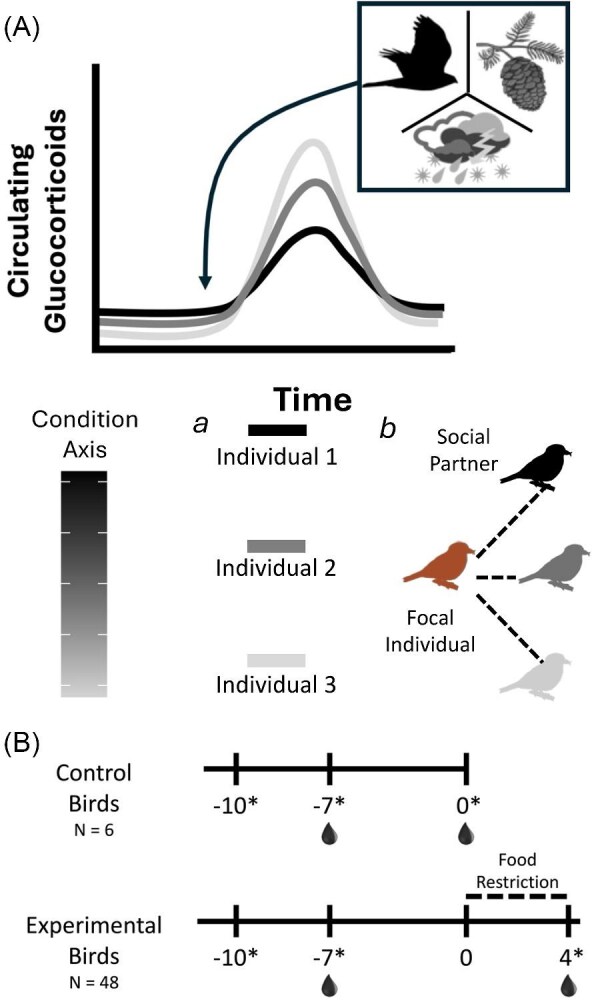
**(A)** Conceptual figure illustrating the differential modulation of the increase in circulating glucocorticoids following an environmental challenge (e.g., changes in food availability, predation attempt, or inclement weather) based on differences in intrinsic state or social environment. Interpreting the graph using legend *a*, lines depict the glucocorticoid response of three individuals that differ in condition as indicated by the line color and condition axis. Using legend *b*, lines depict variable glucocorticoid responses based on the condition of social partners, wherein darker or lighter colors indicate social partners with higher or lower condition values, respectively. The scale of the *x* and *y*-axis of the graph are not specified to capture the potential for acute or chronic elevation of circulating glucocorticoids in response to short term (e.g., predation attempt) or prolonged (e.g., food shortage or inclement weather) environmental challenges. **(B)** Study timeline describing measurement and sample collection relative to the food restriction period for control and experimental groups. For both groups, blood sampling took place 3 days after birds were moved to acoustic chambers (i.e., Day -7). The second samples collected from control birds occurred 10 days after birds were moved into acoustic chambers (i.e., Day 0) to assess the effects of the study protocol on birds’ body condition and glucocorticoid physiology prior to the food restriction. For the experimental birds, the beginning of the food restriction occurred on Day 0, 10 days after entering the chambers, and the second sample collection occurred following 4 days of food restriction (i.e., Day 4). Asterisks in figure indicate days when body condition measurements were collected, and droplets denote days blood samples were collected. Numbers indicate day of study relative to the start of the food restriction.

Glucocorticoids and the neuroendocrine pathway associated with their production (i.e., the hypothalamic-pituitary-adrenal/interrenal axis, hereafter the HPA axis) are evolutionarily conserved mediators of physiological and behavioral responses to environmental challenges (Sapolsky 2000; Wingfield 2013; Romero and Gormally 2019). At circulating concentrations associated with normal circadian and seasonal variability (i.e., predictive homeostasis in Romero et al. 2009), glucocorticoids mediate multiple physiological and behavioral processes, particularly those associated with energetic and metabolic pathways, to meet the demands of predictable environmental changes or life history stage transitions (Landys et al. 2006). In response to unpredictable challenges, glucocorticoids are often elevated to promote a suite of behavioral and physiological changes that maintain the stability of internal systems and promote recovery from environmental challenges (Fig. 1A; Landys et al. 2006; Romero et al. 2009; Taborsky et al. 2022). Though such glucocorticoid-mediated responses facilitate overcoming environmental challenges, they can also be costly depending on the nature of the challenge and, more broadly, the state of the organism (Taborsky et al. 2022).

Considerable variation often exists amongst individuals and sampling contexts in glucocorticoid physiology (Sapolsky 2000; Ellis et al. 2006; Hau et al. 2016). Such variation has been well characterized experimentally by differences in circulating concentrations in the absence of acute handling stress and the maximum concentration reached following standardized capture and restraint (Taff et al. 2018; Taff et al. 2022). Individual differences in the temporal dynamics of the glucocorticoid response to acute stressors can have fitness consequences (Breuner et al. 2008; Bonier et al. 2009; Romero et al. 2009; Taff and Vitousek 2016), particularly associated with survival (Romero and Wikelski 2001; Blas et al. 2007; Angelier et al. 2009; Crespi et al. 2013). Increases in glucocorticoids and the stress response more broadly can also directly induce oxidative stress and pathological tissue and DNA damage (Costantini et al. 2011; Hara et al. 2011; Angelier et al. 2018; Majer et al. 2019; Gormally et al. 2020; Beattie et al. 2024). Consequently, it is hypothesized that circulating glucocorticoids are modulated to prevent damage accumulation (Romero et al. 2009; Wada 2019).

A key prediction of life history theory is that organismal function, including responses to environmental challenges, should be modulated based on an individual’s residual reproductive value (i.e., expected remaining reproductive output) (Stearns 1992; McNamara and Houston 1996; Wingfield and Sapolsky 2003; Schoenle et al. 2018). Residual reproductive value declines with age in many species and a number of studies have demonstrated how glucocorticoid physiology can vary with chronological age (Heidinger et al. 2006; Wilcoxen et al. 2011; Elliott et al. 2014; Lendvai et al. 2015; Angelier et al. 2020). Age-related variation in glucocorticoid physiology can arise due to the selective disappearance of individuals with certain glucocorticoid phenotypes (Wilcoxen et al. 2011) or the down regulation of the glucocorticoid response to unpredictable challenges amongst individuals with a low residual reproductive value (Wingfield and Sapolsky 2003; Heidinger et al. 2006). Further, in some cases, age-related variation is only revealed in specific contexts [e.g., (Angelier et al. 2020)]. The aging process is, however, heterogeneous and individuals of the same chronological age can vary in biologically meaningful ways due to differences in both their exposure to and ability to cope with environmental challenges (Yashin et al. 1985). Indices that more effectively capture individual differences in condition or physiological state are therefore also highly relevant state variables for understanding variation in glucocorticoid physiology.

Two indices that capture individual differences in condition are body condition (Peig and Green 2010), an index of metabolic state and energy reserves, and telomere length (Shay and Wright 2019). Across broad taxonomic scales, body mass has been found to negatively covary with circulating glucocorticoids (Vitousek et al. 2019), though the relationship is often context-specific and bidirectional within species. For instance, elevated glucocorticoids can support behaviors that facilitate mass gain when foraging opportunities are available (Astheimer et al. 1992; Breuner et al. 1998; Schoenle et al. 2018). When faced with starvation, however, increases in glucocorticoids mobilize energy stores through catabolic pathways to support the internal systems needed to survive (Cherel et al. 1988; Romero and Wikelski 2001). Further, telomeres are found at the end of linear chromosomes and are composed of a protein complex and repeated sequences of DNA. Functionally, telomeres maintain chromosomal integrity and are involved in a variety of cellular signaling processes (Shay and Wright 2019). Glucocorticoids have been proposed to mediate telomere dynamics [i.e., within-individual changes in telomere lengths; (Angelier et al. 2018)] and greater telomere attrition during development has been found to be associated with lower stress-induced glucocorticoids (Andrews et al. 2017). Environmental challenges promote telomere shortening (Hau et al. 2015; Salmón and Burraco 2022) and shorter telomeres are often associated with reduced survival and lifespan (Heidinger et al. 2012; Wang et al. 2018). The extensive among individual variation in telomere lengths, even amongst those of the same chronological age, is therefore proposed to reflect, in part, individual differences in the accumulation of wear and tear (Boonekamp et al. 2013; Tobler et al. 2022; Monaghan 2024). Viewed together, differences in body condition and telomere lengths represent potentially important variation in state that can relate to individual glucocorticoid physiology.

The Red Crossbill (*Loxia curvirostra*) is a gregarious, nomadic songbird that specializes on foraging for conifer seeds (Benkman and Young 2020). The movement and breeding behavior of Red Crossbills is dependent on the local availability of conifer seeds (Benkman and Young 2020), an ephemeral resource that can be produced in large quantities during spatially unpredictable, but locally synchronous, periods of conifer cone production [i.e., masting events; (LaMontagne et al. 2020)]. Red Crossbills can make irruptive movements out of their normal range when conifer seeds are scarce and can initiate breeding when seeds are abundant outside of the molting period (Koenig and Knops 2001; Benkman and Young 2020). Information acquired from conspecifics has been found to improve foraging efficiency in Red Crossbills (Smith et al. 1999) and promote adaptive physiological responses to subsequent declines in food availability (Cornelius 2022). Though the specific nature of the cues conveying this information remains to be determined, experimental evidence does demonstrate that individuals are selective in their use of social information (Mori et al. 2025). Additionally, reduced food availability causes an increase in circulating glucocorticoids, and food restricted birds with food restricted neighbors exhibit a remodeling of the HPA axis that involves relatively greater increases in circulating glucocorticoids and changes in the expression of neural receptors for glucocorticoids in the hippocampus and the paraventricular nucleus (Cornelius et al. 2010, 2018). Importantly, these changes in glucocorticoid function were not present in birds without food restricted neighbors (Cornelius et al. 2010, 2018). Food availability is therefore an ecologically relevant source of environmental variation and social cues mediate behavioral and physiological processes associated with foraging and responses to food scarcity in Red Crossbills.

Here, we studied Red Crossbills to test hypotheses explaining sources of among-individual variation in glucocorticoid physiology (Fig. 1A). We measured circulating corticosterone, the main avian glucocorticoid, before and during food restriction in isolated pairs of Red Crossbills composed of a juvenile and adult. At each sampling time point, individuals were sampled immediately after capture and following 30 min of standardized capture and restraint. We refer to these two sampling time points as baseline and stress-induced glucocorticoids, respectively. We consider baseline glucocorticoids to reflect the current physiological state of the organism whereas stress-induced glucocorticoids reflect the organismal response to an acute perturbation (Landys et al. 2006). We use a food manipulation protocol which previously demonstrated food availability and social cues interact to modulate glucocorticoid function in Red Crossbills (Cornelius et al. 2010, 2018). We quantified support for two hypothesized sources of individual variation in glucocorticoid physiology: body condition and telomere lengths. Further, given that social cues mediate glucocorticoid function and organismal responses to food restriction in Red Crossbills (Cornelius et al. 2010, 2018; Cornelius 2022), we hypothesized that social partner body condition and social partner telomere lengths mediate the known effects of social cues on Red Crossbill glucocorticoid physiology. These two hypotheses related to the social environment reflect the potential that partner condition, as measured by an index of metabolic state (i.e., body condition) or an index of wear and tear (i.e., telomere length), influences the nature of or response to social cues from that partner (Vernasco et al. 2022). If intrinsic or social factors shape glucocorticoid physiology, circulating glucocorticoid levels should covary with the relevant predictor (e.g., body condition or telomere length of the individual or its partner). Further, our study design allows us to test if such associations are independent or dependent on the presence of an ecologically relevant challenge (i.e., food availability for Red Crossbills). By examining connections between different levels of biological organization, from cellular traits, endocrine function, and the social environment, our study provides an integrative perspective on why individuals exhibit different responses to ecologically relevant environmental challenges.

## Methods

### Animal capture and housing

In September and October 2021, 56 wild Red Crossbills (ecotype 3, Benkman and Young 2020) were captured at multiple sites within Oregon and Washington. Birds were aged as either juveniles (i.e., <1 year old) or adult (i.e., 1+ years old) using plumage and morphology as previously described (Pyle 1997). Birds were sexed using plumage characteristics (Pyle 1997) and juvenile Red Crossbills were genetically sexed by the Washington Animal Disease Diagnostic Laboratory using the methodology described in Çakmak et al. (2017) and DNA from blood samples. After capture, birds were transported to Washington State University and housed in one of two outdoor aviaries (*n* = 27 and 29 individuals per aviary) and provided a diet of whole sunflower seeds (i.e., with shells), sunflower hearts, and Roudybush Small Bird Maintenance Diet (Woodland, CA, USA; hereafter Roudybush). All birds had *ad libitum* access to water and grit prior to and during all study procedures. For full details related to animal capture and housing see Supplementary Material Section 1. Birds were collected under scientific permits from the US Fish and Wildlife Service, Washington Department of Fish and Wildlife, and Oregon Department of Fish and Wildlife. All procedures were approved by the Washington State University Institutional Animal Use and Care Committee.

### Study design

Our study design replicated the food restriction protocol used in (Cornelius et al. 2010, 2018), which generated socially-mediated changes in HPA axis function. These previous studies demonstrate the consistent effect of the food restriction on Red Crossbill physiology and behavior as well as socially mediated changes in circulating glucocorticoids and their receptors by comparing experimental birds to a control group which did not receive the food restriction. Given the effects of the food restriction are well described, we did not include a similar control group in the current study. Previous studies using this experimental design did not measure stress-induced glucocorticoids, but we nonetheless assume that the observed effects on stress-induced glucocorticoids are due to the food restriction.

The current study occurred between December 27th, 2021 and March 7th, 2022. The procedure began by moving randomly selected pairs consisting of one adult and one juvenile Red Crossbill (*n* = 54 individuals, 27 pairs) into separate, but immediately adjacent, cages within a single acoustic chamber (Lafayette Instrument). Adult-juvenile pairs were used to standardize the age composition of pairs. Each bird therefore experienced the following environmental manipulation while being housed individually and adjacent to their social partner. Individuals were paired using a stratified random approach wherein birds were first grouped by sex and then randomly assigned a same-sex social partner of the opposite age class (*n*_male-male pairs_ = 13, *n*_female-female pairs_ = 9). Due to a limited number of adult females, 5 pairs were composed of an adult male and a juvenile female. Six acoustic chambers were available, requiring five groups of 12 birds each. Analysis of differences in body condition and circulating corticosterone between each type of cage sex ratio revealed no significant differences between the three groups within the data (Supplementary Fig. 1).

Each bird's body condition was measured, as described below, before moving them into the acoustic chambers. Birds were moved into acoustic chambers to limit the social cues to only those provided by social partners. While in acoustic chambers, maintenance and care for birds was conducted daily at 1100. After three acclimation days within acoustic chambers and starting 2 h after the lights turned on, birds experienced a standardized handling and restraint protocol as described in Wingfield et al. (1992). Specifically, a ∼70 uL blood sample was collected within 3 min of capture and then the bird was restrained in a cloth bag (as described below) until a second ∼70 uL blood sample was collected 30 min after the initial capture to measure baseline and stress-induced circulating corticosterone, respectively (baseline mean time from capture to collection = 79.3 s, min = 42 s, max = 160 s; stress-induced mean time from capture to collection = 31.3 min, min = 29.9, max = 34.4 min, mean time of day ± sd = 1000 ± 32 min). Blood samples were collected using heparinized capillary tubes following brachial venipuncture with a 26-gauge needle. During the restraint period, between collecting the baseline and stress-induced blood samples, birds were removed from the cloth bag and a brief series of body condition measurements were quickly collected as described below. Following condition measurements, birds were returned to the cloth bag until the second blood sample was collected and, after the second sample was collected, returned to their cages. Starting the day after the collection of these pre-food restriction blood samples, food intake was measured daily for 6 days by quantifying the mass of the food provided and the mass of food remaining the following day within each cage, during routine care. Birds then experienced one of two protocols (Fig. 1B). The first round of birds (*n* = 3 pairs, 6 individuals) experienced a control protocol that facilitated assessment of the bird’s physiological state immediately prior to the food restriction. This group of birds allowed for an understanding of the effects of housing birds in acoustic chambers and the sampling protocol for measuring body condition and collecting blood samples independent of food restriction. Therefore, 7 days after collecting the initial blood samples, on January 6th, 2022, baseline and stress-induced blood samples were collected from control birds using the standardized capture and restraint protocol described above, and body condition measurements were collected. After the first group of birds, the remaining groups (i.e., 2 through 5, *n* = 24 pairs, 48 individuals) experienced identical conditions with the exception that, starting at 1100, 7 days after collecting the initial blood samples (i.e., January 17th, February 1st, February 16th, or March 3rd, 2023), birds were provided with 75% of their average food intake, provided as a daily allotment, for 94 h. We chose to provide 75% of an individual’s average food intake because this manipulation mimics the experimental design previously used and is therefore known to be a mild food restriction that elicits a known response (Cornelius et al. 2010, 2018). Hereafter, we refer to the group of birds that did not experience the food restriction (i.e., the first group of birds) as control birds and the group of birds that did experience the food restriction as experimental birds. After 94 h of the restricted food protocol and on Day 4 of the food restriction, baseline and stress-induced blood samples were collected and body condition was assessed using the methods described below. We refer to the sampling point after 94 h of food restriction as “post-food restriction.” After sampling, birds were restored to *ad libitium* food. One juvenile male was found to be in poor health 72 h into the food restriction and was immediately provided food *ab libitum*. Post-food restriction blood samples were not collected for this individual, though post-food restriction condition data (measured on Day 4) indicated it was 3.2 g lighter and its fat score decreased from 6 to 3. Data from this individual’s social partner were included in subsequent analyses as this bird still exhibited a representative decline in condition over the course of the food restriction.

### Body condition measurements

Body condition was assessed using measurements of body mass, furcular and abdominal fat depots, and pectoralis muscle size as described in Vernasco et al. (2022). Body mass was measured to the nearest 0.01g using an electronic balance. Subcutaneous furcular and abdominal fat depots were visually scored from zero (no fat) to five (bulging fat) and the two scores were summed to generate a single fat score (Wingfield and Farner 1976). Pectoralis muscle size was visually scored from 0 (muscle concave with keel very prominent) to 3 (muscle bulging over keel). All measurements were collected by BJV. The observer was aware of the experimental stage (i.e., before or during food restriction), but blind to previous measurements and telomere lengths. Two of the condition metrics, one being subjective (fat score) and the other objective (body mass), were very highly correlated and the correlation was independent of the experimental stage, providing no evidence of observer bias in condition measurements (Supplementary Fig. 2). A body condition index was generated by scaling all three variables and then ordinating the three variables using principal components analysis with the *princomp()* function in Program R (R Core Team 2022). All three variables loaded positively onto the first principal component (loadings range = 0.49–0.63), which explained 77% of the variation and was subsequently used as the body condition index. A size-corrected body mass measurement (calculated using tarsus length as described in Peig and Green 2010) was not used because the two body condition indices (i.e., corrected for structural size and uncorrected) were highly correlated (Pearson correlation coefficient = 0.93) and using the size-corrected measure did not affect the results of the statistical analysis.

### Blood sample processing and corticosterone assay

Immediately following collection of blood samples, capillary tubes containing collected blood were sealed with Critoseal^®^ and stored on ice. Plasma was separated from red blood cells by centrifuging samples for 5 min at 10,000 rpm (average plasma volume = 27.4 uL, range = 17–39 uL). Plasma was stored at −20°C until being shipped on dry ice to Virginia Tech. Red blood cells were added to microcentrifuge tubes containing 1 mL of 100% ethanol, a storage buffer known to adequately preserve DNA for telomere analyses (Eastwood et al. 2018), and stored at −20°C. For both the corticosterone and relative telomere length assays, samples from the same individual were grouped together in random order and then these individual groups were randomly ordered. Three samples were lost during centrifugation, two were post-food restriction, stress-induced samples collected from a juvenile male and adult female. The third was a baseline sample collected from an adult male post-food restriction.

Total plasma corticosterone concentrations were quantified following extraction with dichloromethane (average extraction efficiency [min, max]: 73.81% [60.91–84.05]) using three separate direct radioimmunoassays (Wingfield et al. 1992; Beck et al. 2016). Samples were analyzed in duplicate and concentrations were adjusted for each sample’s extraction efficiency and original plasma volume. Standards were used to calculate the intra-assay coefficient of variation and the inter-assay coefficient of variation. The intra-assay coefficients of variation were 4.6, 3.1, and 5.3% and the inter-assay coefficient of variation was 11.7%. The average detection limit for the assay was ∼1.5 ng/mL, and all measured samples that fell below the assay’s detection limit were assigned the plasma-volume and extraction efficiency corrected detection limit for that sample. Sample concentrations were measured in ng/mL and log-transformed prior to statistical analyses to meet the normality assumption.

### Relative telomere length measurement

DNA was extracted from red blood cells stored in 100% ethanol using a Gentra Puregene Blood Kit (Qiagen) and a modified extraction protocol as described in Vernasco et al. (2021). DNA purity and concentration were assessed using a NanoDrop ND-1000 (mean DNA concentration ± SD = 226.1 ± 139.9 ng/uL, range of DNA concentrations = 26.1–823.9 ng/uL, mean 260/280 ratio ± SD = 1.85 ± 0.03, mean 260/230 ratio ± SD = 1.92 ± 0.29) and high DNA integrity was confirmed using gel electrophoresis. Relative telomere lengths were then quantified using real-time quantitative polymerase chain reaction (PCR) (qPCR) following the methodologies described in Criscuolo et al. (2009), Eastwood et al. (2018), and Vernasco et al. (2021). Relative telomere lengths are quantified using this approach by comparing amplification kinetics of telomere repeats to those of a single-copy gene, in this case glyceraldehyde-3-phoshate dehydrogenase (GAPDH). The GAPDH primers were developed from the GAPDH sequence of a closely related species (*Spinus pinus*, NCBI KT358792) using the primer design tool in Geneious v10.2.3 (Kearse et al. 2012). Cycle quantification values and individual well qPCR efficiencies for samples were calculated using *LINREGPCR* version 11 (Ruijter et al. 2009). Relative telomere lengths (hereafter, telomere length) were calculated following equation one in Pfaffl (2001). Technical repeatability was calculated using measurements of technical replicates of each sample and inter-plate repeatability was measured using replicate measurements of a randomly selected subset of samples that were included on each qPCR plate. Technical repeatability of rTL measurements was estimated to be 0.94 (95% CIs [0.92, 0.95], *P* < 0.001) and inter-plate repeatability of rTL measurements was estimated to be 0.88 (95% CIs [0.47, 0.96], *P* < 0.001). For a more detailed description of the telomere length measurements see Section 2 of the Supplementary Material.

### Statistical analyses

Here, we use a well-established food-manipulation protocol and longitudinal sampling to understand sources of individual variation in glucocorticoid physiology (i.e., body condition, telomere lengths, and social partner traits) and if such relationships depend upon environmental context (i.e., food availability). Studying Red Crossbills necessitates the use of wild caught birds, resulting in a sample population that exhibits group-level differences (i.e., age and sex) that could influence the relationship between glucocorticoid physiology and condition variables. Indeed, glucocorticoid function is well known to differ with age (Heidinger et al. 2006; Wilcoxen et al. 2011; Elliott et al. 2014; Lendvai et al. 2015; Angelier et al. 2020) and sex (Wingfield et al. 1995; Quinn et al. 2014) and patterns of covariation between circulating glucocorticoids and individual condition could therefore vary by either group variable. To quantify support for these group differences within our sample population and make evidence-based analytical decisions, we used a hypothesis-informed multi-stage, build up modeling process (Morin et al. 2020). Specifically, statistical support for group-level differences in glucocorticoid physiology was first quantified using Akaike’s information criterion (AIC; see Table 1 and *Assessment of Age and Sex Differences* for more details) and statistical support for the hypotheses of interest to this study (Table 2A) was then quantified within each identified group, again with AIC (see *Analysis of Individual Variation in Glucocorticoid Physiology* for more details). By first identifying group differences and then examining the patterns of individual differences within groups, our approach limits the number of interactions considered in each model to one. Indeed, there are multiple issues associated with including more than 1 interaction term in an individual model (e.g., large model sets, variance inflation, and complicated interpretations of model effects), particularly with the sample size of the current study. Limiting the number of interaction terms also facilitates concise (i.e., 4–20 models total), hypothesis driven model sets as recommended by Burnham and Anderson (2004). Lastly, our approach also prioritizes parsimony in model structure as AIC penalizes models with more complexity and supports those with more parsimonious covariate combinations.

**Table 1:** Model tables associated with analysis of changes in log-transformed, baseline and stress-induced CORT in experimental birds. Model selection results are displayed in Table 1A. Type is a categorical variable denoting baseline or stress-induced CORT, context is a categorical variable denoting pre- or post-food restriction, and age is a categorical variable denoting juvenile or adult. The summary of the top supported model is shown in Table 1B. The reference group for this model is juvenile, baseline, and pre-food restriction values. Pairwise comparisons, calculated from the top supported model, are shown in Table 1C.

**1A** CORT AIC table (*n* = 186)					
Model	Params	ΔAICc	Weight		
Type * Context * Age	10	0	0.83		
Type * Context	6	3.77	0.13		
Type * Context + Age	7	7.63	0.02		
Type * Context + Sex	7	7.81	0.02		
Type * Context + Sex + Age	8	11.61	0.00		
Type * Context * Sex	10	14.59	0.00		
Type * Context * Sex * Age	18	16.01	0.00		
**1B** Summary of Top-supported Model—condition/marginal *R*^2^ = 0.74/0.6					
**Variable**	**Estimate**	**SE**	**df**	***t*-value**	***P*-value**
Intercept	0.54	0.15	131.40	3.57	0.001
Stress-induced	2.32	0.17	132.31	13.42	<0.000001
Post-food restriction	0.90	0.18	133.34	5.14	0.000001
Age:Adult	0.53	0.22	133.26	2.43	0.02
Stress-induced * Post-food restriction	−0.93	0.25	133.04	−3.72	0.0003
Stress-induced * Adult	−0.25	0.25	132.64	−1.01	0.31
Post-food restriction * adult	−0.61	0.25	133.15	−2.45	0.02
Stress-induced * Post-food restriction * Adult	−0.17	0.35	132.98	−0.48	0.63
**1C** Pairwise Comparisons					
	**Estimate**	**SE**	**df**	***t*-ratio**	***P*-value**
Baseline contrasts					
Pre-food restriction juvenile—post-food restriction juvenile	−0.90	0.18	133.12	−5.14	0.00001
Pre-food restriction juvenile—Pre-food restriction adult	−0.53	0.22	133.04	−2.43	0.03
Pre-food restriction juvenile—post-food restriction adult	−0.82	0.21	131.17	−3.82	0.001
Post-food restriction juvenile—pre-food restriction adult	0.38	0.22	134.70	1.72	0.11
Post-food restriction juvenile—post-food restriction adult	0.08	0.22	132.89	0.38	0.71
Pre-food restriction adult—post-food restriction adult	−0.30	0.18	132.74	−1.68	0.11
Stress-induced contrasts					
Pre-food restriction juvenile—post-food restriction juvenile	0.03	0.18	134.47	0.17	0.86
Pre-food restriction juvenile—pre-food restriction adult	−0.28	0.21	131.17	−1.29	0.24
Pre-food restriction juvenile—post-food restriction adult	0.53	0.22	133.04	2.45	0.05
Post-food restriction juvenile—pre-food restriction adult	−0.31	0.22	136.94	−1.39	0.24
Post-food restriction juvenile—post-food restriction adult	0.50	0.22	138.62	2.24	0.05
Pre-food restriction adult—post-food restriction adult	0.81	0.18	132.74	4.60	0.0001

**Table 2:** Hypotheses (A) and model selection results (B–D) for analyses examining sources of variation in circulating corticosterone in juvenile and adult Red Crossbills

**2A** Models and associated hypotheses				
Model	Hypothesis			
Context + rTL	Circulating corticosterone depends on individual or partner telomere lengths, independent of food availability			
Context + body condition	Circulating corticosterone depends on individual or partner body condition, independent of food availability			
Context * rTL	The relationship between circulating corticosterone and individual or partner telomere lengths depends on food availability			
Context * body condition	The relationship between circulating corticosterone and individual or partner body condition depends on food availability			
**2B** Juvenile baseline CORT, individual predictors (*n* = 47)				
**Model**	**Params**	**ΔAICc**	**Weight**	***R* ^2^**
Context * Body condition	6	0.00	1.00	0.65/0.59
Context * rTL	6	17.75	0.00	0.32/0.31
Context + Body condition	5	18.43	0.00	NA/0.33
Context	4	19.11	0.00	NA/0.24
Context + rTL	5	20.15	0.00	NA/0.26
**2C** Adult stress-induced CORT, individual predictors (*n* = 46)				
**Model**	**Params**	**ΔAICc**	**Weight**	***R* ^2^**
Context + rTL	5	0.00	0.53	0.79/0.3
Context * rTL	6	0.41	0.43	0.73/0.19
Context	4	5.58	0.03	0.73/0.18
Context + Body condition	5	10.38	0.00	0.73/0.19
Context * Body condition	6	14.10	0.00	0.73/0.2
**2D** Juvenile baseline CORT, social partner predictors (*n* = 46)				
**Model**	**Params**	**deltaAICc**	**Weight**	***R* ^2^**
Context * Partner rTL	6	0.00	0.74	NA/0.34
Context + Partner rTL	5	3.42	0.13	0.37/0.28
Context	4	3.70	0.12	NA/0.24
Context + Partner body condition	5	7.97	0.01	NA/0.25
Context * Partner body condition	6	11.71	0.00	NA/0.25

*Note:* Circulating corticosterone was log-transformed before analysis and predictor variables were either measured from focal individuals to examine intrinsic influences (B, C) or social partners (D). Model tables in which the top supported model was the null model are not shown. All AICc tables associated with individual and social partner predictor variables can be found in Supplementary Tables 6 and 8, respectively. *R*^2^ values represent conditional (variance explained by fixed and random effects) and marginal (variance explained by fixed effects) values respectively. Conditional *R*^2^ with NA values represent estimates wherein the variance explained by random effects equals zero.

All statistical analyses were conducted in R version 4.2.0 (R Core Team 2022). All linear mixed models were built using the *lme4 package* v 1.1–29 (Bates et al. 2015) and *P*-values were generated using the *lmerTest* package v 3.1–3 (Kuznetsova et al. 2017). All model diagnostics were performed by evaluating the output from the *check_model* function in the *performance* package v 0.9.0 (Lüdecke et al. 2021). For analyses focused on experimental birds, a random effect of the experimental round was initially included in all models but was subsequently removed as it explained little to no variance in response variables. Including a random effect of dyad identity in the analysis focused on identifying group differences (e.g., age and sex differences) in corticosterone also explained no variation and was subsequently removed. Results also did not change following the removal of this random effect. Including a fixed effect of experimental round in analyses of group differences in body condition and circulating corticosterone also revealed no significant differences between experimental rounds. Marginal and conditional *R*^2^ was calculated for models analyzing circulating corticosterone using the *r*2() function in the performance package (Lüdecke et al. 2021). The marginal *R*^2^ quantifies the amount of variance explained by fixed effects and the conditional *R*^2^ represents the amount of variation explained by fixed and random effects.

#### Analysis of control bird body condition and corticosterone

To understand the effects of the procedure outside of those related to the food restriction (e.g., housing in acoustic chambers), changes in body condition and circulating corticosterone were analyzed amongst control birds using two linear mixed models. The model examining changes in control bird body condition included an effect of sampling context (i.e., Day -10/pre-chamber, Day -7/post-chamber, and Day 0/pre-food restriction). The model examining changes in circulating corticosterone included an interaction between sampling context and the type of sample (i.e., baseline or stress-induced corticosterone). In both models, individual identity was included as a random effect. No other covariates were included in these analyses due to limited sample sizes (i.e., *n* = 18 condition measurements, 24 corticosterone measurements), though plots of data by age and sex do not suggest strong effects of these covariates (Supplementary Figs. 3 and 4).

#### Analysis of experimental bird body condition and telomere length

To quantify sex- and age-specific changes in body condition and telomere lengths amongst experimental birds, a linear mixed model that included an interaction between fixed effects denoting sampling context (i.e., Day -10/pre-chamber, Day -7/pre-food restriction, and Day 4/post-food restriction), age, and sex and a random effect denoting individual ID was used. A random effect of plate was also included in the telomere length model. For models with significant effects, estimated marginal means and their 95% confidence intervals for each group at each sampling context were calculated using the *emmeans* function within the *emmeans* package v 1.7.4 (Lenth 2022). Post-hoc comparisons were conducted using simple contrasts with the *emmeans* package to examine changes in body condition over the course of the study and the extent to which changes in body condition were dependent on age or sex.

Any observed age- or sex-specific effects of the food restriction on body condition may be attributed to age or sex differences in initial food intake, which could lead to the food restriction being relatively more severe for a particular group. For instance, if one age or sex class exhibits higher food intake, then a 25% reduction in food intake would be a greater amount of food not being provided for that group relative to other groups. Examining the relative decrease in mass loss could provide insight into whether a particular age or sex group was more severely affected by the food restriction. To test these possibilities, we examined if either food intake or relative body mass loss differed between the age and sex classes using linear models. For full details on this analysis see Section 3 of the Supplementary Material.

#### Analysis of age and sex differences in glucocorticoid physiology

To understand how the food restriction influenced baseline and stress-induced circulating corticosterone over the course of the study and identify relevant group differences, circulating corticosterone values were first log-transformed to normalize the data and 7 models that included different additive and interactive, fixed-effects of age and sex were compared using AICc-based model comparisons (Table 2A, Burnham and Anderson 2004). A random intercept term denoting individual ID was also included, though a random slope term was not included due there being only 2 repeated measures. The null model in these comparisons contained no sex or age effects and was used as the reference model to assess relative support for more complex models. If the top-supported model included effects of age or sex, estimated marginal means and their 95% confidence intervals for each group at each sampling context were calculated using the *emmeans* function within the *emmeans* package. Simple contrasts were used to compare the estimated marginal means such that only baseline and stress-induced values were compared to other baseline and stress-induced values, respectively. *P*-values were adjusted based on the number of comparisons using the false-discovery rate adjustment (i.e., by setting the adjust argument to “fdr"). We did not examine sources of variation related to the difference in baseline and stress-induced corticosterone (e.g., delta-corticosterone or fold change in corticosterone) because corticosterone interacts with different receptors at baseline and stress-induced concentrations. As such, the biological relevance of the difference between baseline and stress-induced corticosterone is not known [See Box 1 in (Romero 2004)].

#### Analysis of individual variation in glucocorticoid physiology

To quantify support for hypotheses explaining individual variation in circulating corticosterone amongst experimental birds (Table 2A), linear mixed models and the AICc-based model selection approach described in Burnham et al. (2011) was used. Baseline and stress-induced corticosterone were examined using separate model sets due to known differences in corticosterone concentrations associated with the physiological responses to the capture and restraint protocol (Wingfield et al. 1992). Age- and sex-specific corticosterone concentrations were also analyzed using separate model sets if the prior analyses (see *Assessment of Age and Sex Differences* for more details) identified age- or sex-related differences in either baseline of stress-induced CORT. In all model sets, the null model included a variable denoting sampling context (i.e., pre- or post-food restriction) and a random effect of individual ID. Support for models ranked above the null model (i.e., with a lower AICc value) was examined using the ΔAICc values and model weight (Burnham et al. 2011). Additional models included either additive or interactive effects of body condition and telomere length, either of the focal individual or its social partner (Table 2A). For these analyses, measurements of condition and relative telomere length were matched with the corresponding corticosterone measurements collected at the same time point (i.e., pre-food restriction or post-food restriction). For analyses of individual variation in circulating corticosterone, multiple explanatory variables were not included in a single model to simplify interpretations of results and due to the limited sample size. When model selection uncertainty was present (i.e., multiple models within 2 ΔAICc of the top-supported model), models were examined for uninformative parameters by calculating the 85% CIs for each predictor, and parsimony was prioritized (Arnold 2010).

Due to known effects of social cues on glucocorticoid physiology in Red Crossbills (Cornelius et al. 2010, 2018), the above modeling procedure was replicated using explanatory variables derived from measurements from an individual’s social partner (i.e., the other individual in the acoustic chamber). This second analysis allowed us to examine the extent to which the traits of an individual’s social partner (i.e., the social environment) contribute to individual variation in either baseline or stress-induced circulating corticosterone. Slope estimates were generated for the top supported models using the *emtrends* function in the *emmeans* package.

Repeatability estimates were calculated for both condition variables and baseline and stress-induced corticosterone using the rptR package v 0.9.22 (Stoffel et al. 2017). Both indices of condition were significantly repeatable (*R* > 0.4 for all variables, Supplementary Table 1). Plots displaying the correlations between explanatory variables and their distributions can be found in Supplementary Fig. 2.

If an effect of body condition was detected, we used the within-individual centering technique described in van de Pol and Wright (2009) as a post-hoc analysis to understand if the relationship was driven by between-individual differences in body condition or within-individual changes in body condition that were associated with the food restriction. For this analysis, the between-individual effect was calculated by averaging the three measurements of body condition recorded during the study (i.e., pre-chamber, pre-food restriction, and post-food restriction) and the within-individual effect was calculated by subtracting an individual’s average body condition from the measured body condition value. An interaction between the sampling context (i.e., pre- or post-food restriction) and body condition metrics was included to determine if the relationship between baseline corticosterone and body condition depended on the food restriction.

## Results

### Analysis of control bird body condition and corticosterone

Control birds exhibited no significant changes in body condition over the course of the study, which did not include food restriction (β_pre-chamber_ − _post-chamber_ = 0.27, *P* = 0.54; β_post-chamber_ − _pre-food restriction_ = 0.02, *P* = 1, Supplementary Fig. 3, Supplementary Table 2A, B). Baseline corticosterone exhibited a significant decline between the two blood sampling events (β_post-chamber_ − _pre-food restriction_ = −0.39, *P* = 0.02), while stress-induced corticosterone values did not differ between sampling events (β_post-chamber_ − _pre-food restriction_ = −0.05, *P* = 0.71; Supplementary Fig. 4, Supplementary Table 2C, 2D).

### Analysis of experimental bird body condition and telomere length

**Fig. 2:**
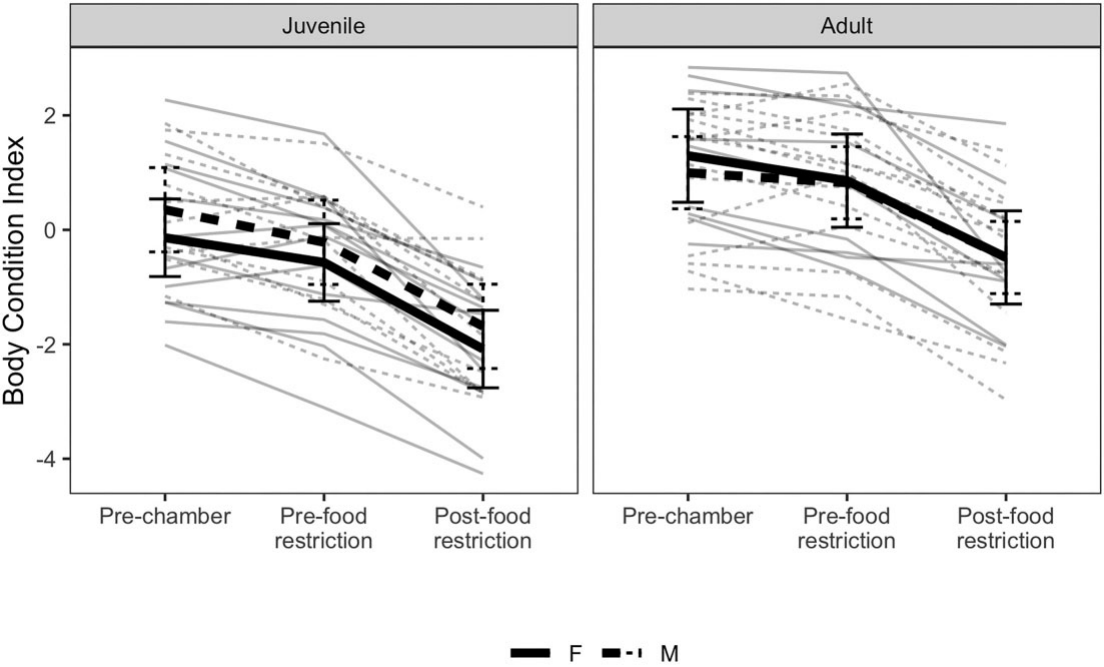
Age and sex differences in the body condition index when experimental birds were moved into acoustic chambers (pre-chamber), after 3 days within acoustic chambers (pre-food restriction) and following 4 days of food restriction (post-food restriction). Thin lines denote individual values, while thick lines connect estimated marginal means from the linear mixed models summarized in Supplementary Table 3. Error bars surrounding estimated marginal means display 95% confidence intervals. Solid lines indicate females while dashed lines indicates males.

Amongst experimental birds, there were no significant age or sex differences in telomere lengths, nor did telomere lengths significantly differ across sampling contexts (Supplementary Table 3A). There were also no sex differences in body condition in either age class at any sampling context (β_F_ − _M_ range −0.49–0.3, *P*-value range 0.33–1; Fig. 2, Supplementary Table 3C). Juvenile (juv) birds had significantly lower body condition than adult (ad) birds at all sampling contexts and this difference was more pronounced amongst males (males: β_juv_ − _ad_ range = –1.61 to –1.43, *P*-value range = 0.004–0.01, females: β_juv_ − _ad_ range = −1.2 to −0.65, *P*-value range = 0.02–0.19; Fig. 2, Supplementary Table 3C). Juvenile females and males exhibited declines in body condition between being transferred to chambers and prior to the food restriction (values are presented F/M: β_pre-chamber_ − _pre-food restriction_ = 0.43/0.57, *P* = 0.01/0.04; Fig. 2, Supplementary Table 3C), while adult females and males did not (F/M: β_pre-chamber_ − _pre-food restriction_ = 0.44/0.18, *P* = 0.37/0.09; Fig. 2, Supplementary Table 3C). Following the food restriction, all age and sex classes exhibited significant declines in body condition that exceeded the declines observed following transfer to the chambers (range β_pre-food restriction_ − _post-food restriction_ = 1.31–1.51, all *P* values <0.0001; Fig. 2, Supplementary Table 3C). Juveniles exhibited significantly higher food intake than adults before the food restriction, independent of sex. Relative body mass loss did not differ between age or sex classes (Supplementary Material Section 3).

### Analysis of age and sex differences in glucocorticoid physiology

**Fig. 3:**
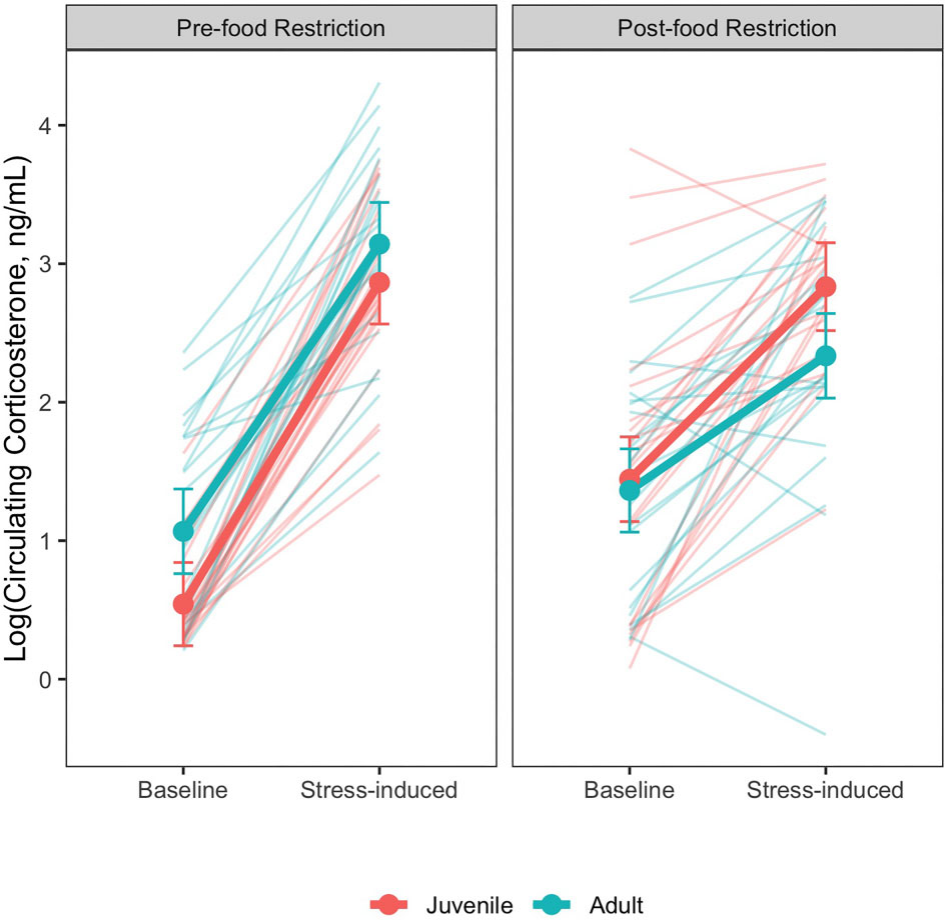
Log-transformed baseline and stress-induced corticosterone values differed with age class both prior to and following food restriction. Thin lines display individual values and thick lines connect estimated marginal means generated from the model summarized in Table 2B. Error bars indicate 95% confidence intervals surrounding estimated marginal means. Color denotes age class.

Stress-induced corticosterone was found to be significantly repeatable (*R* [95% CI] = 0.49 [0.29,0.65]) while baseline corticosterone was not (*R* = 0.10 [0,0.33]). The top supported model explaining variation in circulating corticosterone included interactions between age, sample type (i.e., baseline or stress-induced), and context (i.e., pre- or post-food restriction; Fig. 3, Table 1A, B). Post-hoc pairwise comparisons within age classes subsequently identified that baseline corticosterone was significantly higher post-food restriction in juveniles (β_pre-juv_ − _post-juv_ = −0.87, *P* = 0.00001), while a similar trend was not significant amongst adults (β_pre-Ad_ − _post-Ad_ = −0.30, *P* = 0.13; Fig. 3, Table 1C). Pre-food restriction baseline corticosterone among juveniles was also significantly lower than adult baseline corticosterone pre- and post-food restriction (β_pre-juv_ − _pre-Ad_ = −0.53, *P* = 0.03, β_pre-juv_ − _post-Ad_ = −0.82, *P* = 0.001; Fig. 3, Table 1C). Stress-induced corticosterone levels among juveniles did not differ pre- and post-food restriction (β_pre-juv_ − _post-juv_ = 0.00, *P* = 0.99; Fig. 3, Table 1C). Amongst adults, stress-induced corticosterone significantly decreased following the food restriction (β_pre-Ad_ − _post-Ad_ = 0.81, *P* = 0.00004; Fig. 3, Table 1C). Post-food restriction, stress-induced corticosterone amongst adults was also significantly lower than juvenile stress-induced corticosterone pre- and post-food restriction (β_pre-juv_ − _post-Ad_ = 0.53, *P* = 0.03, β_post-juv_ − _post-Ad_ = 0.53, *P* = 0.03; Fig. 3, Table 1C).

### Analysis of individual variation in glucocorticoid physiology

**Fig. 4:**
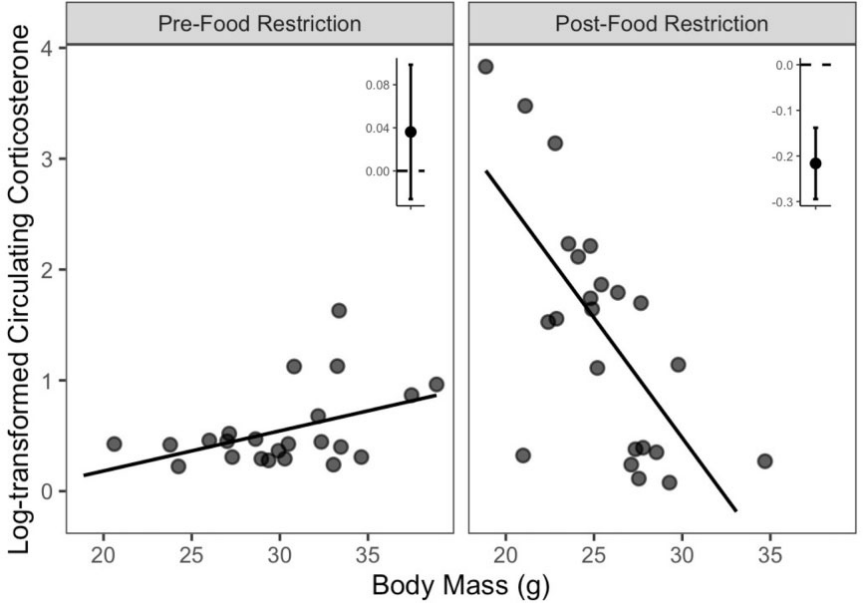
The top-supported model explaining individual variation in baseline corticosterone amongst juvenile Red Crossbills included body condition. The relationship between baseline corticosterone and body condition was dependent on the context (pre- or post-food restriction) and was strongest following the food restriction. Points display individual values, lines represent the line of best fit, and inset graphs display slope estimates and 95% confidence intervals generated by the model summarized in Supplementary Table 4A. See Table 2B for the associated AICc table.

**Fig. 5:**
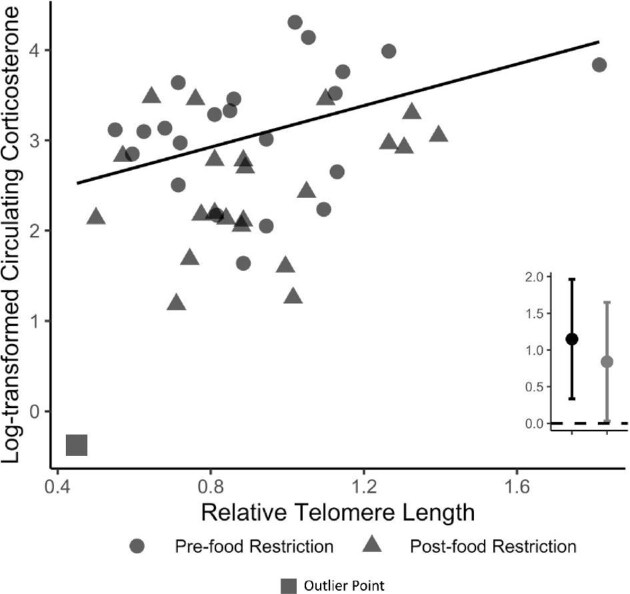
The top-supported model explaining individual variation in stress-induced corticosterone amongst adult Red Crossbills was that with relative telomere length. Though there was an effect of context (pre- or post-food restriction) on adult stress-induced corticosterone, the relationship with telomere length was independent of the context. Points display individual values, line represent the line of best fit, and inset graph displays slope estimate and 95% confidence intervals generated by the model summarized in Supplementary Table 4D. The gray slope value was estimated without the inclusion of the outlier point, which was collected post-food restriction. See Table 2B for the associated AICc table.

Due to the age effects identified above and to simplify model structure, analyses of the relationships between predictor variables and variation in baseline and stress-induced corticosterone were conducted for each age class separately. The top supported model examining variation in juvenile baseline corticosterone included an interaction between body condition and sampling context (Table 2B, Supplementary Table 4A). This model revealed no relationship between body condition and baseline corticosterone pre-food restriction (β_body condition_ [95% CIs] = 0.14 [−0.07, 0.36]), but post-food restriction, there was a negative relationship between body condition and baseline corticosterone (β_body condition_ = −0.66 [−0.88, −0.44]; Fig. 4). The top supported models examining variation in juvenile stress-induced corticosterone and adult baseline corticosterone were the null models (Supplementary Table 8B, C). Amongst models examining adult stress-induced corticosterone, there was equivalent support for two models that included additive or interactive effects of telomere length and sampling context (Table 2C). The more parsimonious model was used for inference as the interaction term was found to be an uninformative parameter (β_rTL * Context_ [85% CIs] = 0.67 [−0.02, 1.38]). The model including additive effects of context and telomere length revealed a positive relationship between stress-induced corticosterone and telomere lengths in adult birds (β_rTL_ = 1.15 [0.33, 1.96]; Fig. 5, Supplementary Table 4D). Further inspection of the relationship revealed the presence of an outlier value, as indicated by the square in Fig. 5. Neither the model selection results, nor the relationship were sensitive to excluding the outlier (β_rTL-no outlier_ = 0.84 [0.03, 1.65], Fig. 5).

As juvenile baseline corticosterone values were found to be dependent on body condition, a post-hoc analysis was conducted to assess the extent to which the relationship was related to between-individual differences and within-individual changes in body condition (van de Pol and Wright 2009). The within-individual centering approach revealed the same context-specific pattern as the initial body condition analysis and, post-food restriction, both between-individual differences and within-individual changes in body condition were negatively associated with baseline circulating corticosterone (β_between-individual_ = −0.64 [−0.89, 0.41], β_within-individual_ = −0.72 [−1.21, −0.21]; Supplementary Fig. 5, Supplementary Table 5A). Equation 3 in van de Pol and Wright (2009) revealed that the post-food restriction, between-individual and within-individual effects were not statistically different in their magnitude (β_between_ − _within_ = 0.07 [−045, 0.59]; Supplementary Table 5B).

**Fig. 6:**
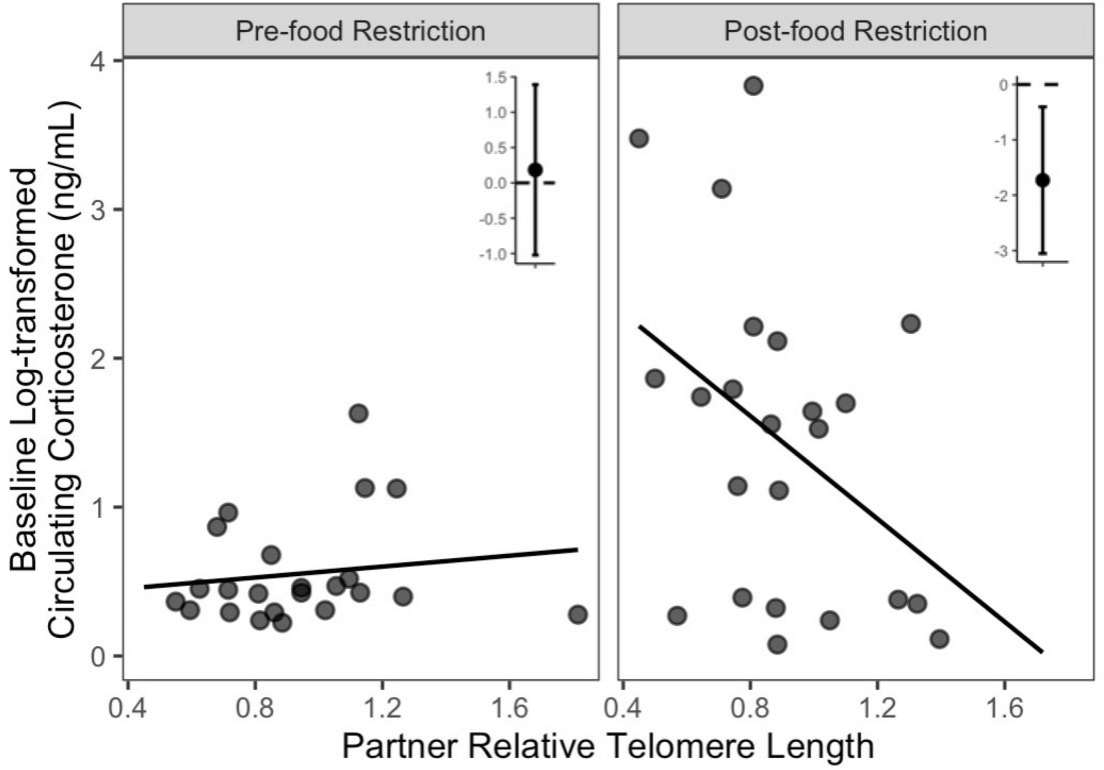
The top-supported social partner model explaining juvenile baseline corticosterone was that with partner relative telomere length. The relationship between baseline corticosterone and partner relative telomere length was dependent on the context (pre-or post-food restriction) and strongest following the food restriction. Points display individual values, lines represent the line of best fit, and inset graphs display slope estimates and 95% confidence intervals generated by the model summary provided in Supplementary Table 7. See Table 6A for the associated AICc table.

Amongst models quantifying support for an effect of social partner traits, the null model (i.e., the model that included only sampling context) was the top supported model in three of the four model sets (juvenile stress-induced, adult baseline, adult stress-induced; Supplementary Table 6). In the model set examining juvenile baseline corticosterone values, the top-supported model included an interaction between the sampling context and the social partner’s telomere length (Table 2C). This model indicated there was no relationship between juvenile baseline corticosterone and partner telomere lengths pre-food restriction (β_partner rTL_ = 0.18 [−1.02, 1.39]), but did identify a significant negative effect of partner telomere length post-food restriction (β_partner rTL_ = −1.73 [−3.05, −0.40]; Fig. 6, Supplementary Table 7). To quantify the probability of observing this effect by chance alone, a follow-up randomization test was conducted. Specifically, partner identities were reassigned at random, and models were re-estimated across 1000 iterations of random partner assignments. Only 3% of the resulting models had a beta coefficient equal to or greater than the observed post-food restriction β_partner rTL_, indicating that there is a low probability of the observed results due to chance alone. Additionally, partner telomere lengths and the body condition of the juvenile focal bird were not correlated (Supplementary Fig. 2A). Including both variables as well as interactions between each and the sampling context in the same model also replicated the results (pre-food restriction: β_partner rTL_ = 0.20 [−0.67,1.08], β_body condition_ = 0.16 [−0.05, −0.37; post-food restriction: β_partner rTL_ = −1.52 [−2.44, −0.59], β_body condition_ = −0.63 [−0.84, −0.43]).

## Discussion

Here, we sample individuals before and after an ecologically relevant challenge that is known to cause socially mediated changes in glucocorticoid function to understand intrinsic and social sources of individual variation in glucocorticoid physiology. Birds exhibited expected declines in body condition and increases in circulating glucocorticoids following the food restriction as identified by studies using an identical experimental design (Cornelius et al. 2010, 2018; Wurtz et al. 2021) and, here, we identify that these changes are dependent on age, but not sex. Specifically, baseline glucocorticoids increased in both age-classes under food restriction. However, only amongst adults did stress-induced glucocorticoids change, with adults exhibiting a decline in concentrations under food restriction. The absence of sex differences is consistent with previous studies of Red Crossbills that have found no sex differences in mass loss following declining food availability (Cornelius 2022, 2024) or in glucocorticoid production across multiple seasons (Cornelius et al. 2012). Further, we also found novel support for hypothesized roles of individual telomere lengths, individual body condition, and the social environment (in the form of partner telomere length) in modulating glucocorticoid concentrations. Some of these relationships were only apparent under food restriction and support for the hypotheses was also variable between the age classes. Amongst adults, stress-induced circulating corticosterone was found to depend on individual telomere lengths, independent of food restriction. Juvenile baseline corticosterone was found to be dependent on body condition and the telomere lengths of their adult social partners, though these relationships were only apparent after the food restriction. Viewed together, our study uses an ecologically relevant environmental challenge to reveal novel relationships between glucocorticoid physiology and individual and social partner condition, as indicated by telomere lengths. These findings highlight the potential for social and condition-related factors to influence glucocorticoid physiology and shed light on state-dependent glucocorticoid modulation and physiological integration more broadly.

The glucocorticoid response to acute stressors is known to be highly flexible and modulated by a number of environmental factors (Schoenle et al. 2018), including food restriction (Romero and Wikelski 2001; Krause et al. 2017). Under the Reactive Scope Model (Romero et al. 2009), maintaining elevated concentrations of glucocorticoids (i.e., within the Reactive Homeostasis range) is costly due to the associated energetic demands, the diversion of resources from self-maintenance functions (e.g., tissue repair), and the diminished ability to continue to cope with challenging conditions. Adaptive modulation of the glucocorticoid response would occur if individual differences in glucocorticoid production were related to the extent of damage accumulation (Romero et al. 2009), but empirical evidence linking individual differences in wear and tear to HPA axis function is limited. Here, we find that adults modulate the corticosterone response to an acute stressor based on their telomere lengths, which are a proposed indicator of wear and tear (Boonekamp et al. 2013, Angelier et al. 2018). Specifically, following the food restriction, stress-induced corticosterone concentrations of adults were reduced and individuals with shorter telomeres exhibited lower concentrations of stress-induced corticosterone both prior to and after the food restriction. This observation suggests that the glucocorticoid response to acute and prolonged environmental stressors can be modulated in a manner that reduces exposure to the pathological effects of elevated glucocorticoids. Indeed, the acute stress-response and glucocorticoid exposure more broadly can induce oxidative stress (Costantini et al. 2011; Majer et al. 2019), DNA damage (Gormally et al. 2020; Beattie et al. 2024), and potentially relate to patterns of telomere shortening (Haussmann et al. 2012; Herborn et al. 2014; Angelier et al. 2018). The observed modulation of the corticosterone response supports the hypothesis that glucocorticoids are adaptively modulated under challenging environmental conditions (i.e., food restriction) and among individuals that have already experienced significant wear and tear, as indicated by their short telomeres (Boonekamp et al. 2013). Whether such adaptive modulation is more prominent in species that must cope with frequent and challenging perturbations, such as Red Crossbills, warrants further investigation. Importantly, the selective disappearance of individuals with high stress-induced corticosterone and short telomere lengths may also contribute to the observed covariation between telomere lengths and stress-induced corticosterone. The age-specific covariation between telomeres and glucocorticoids suggests that juveniles differ in their ability to modulate the HPA axis or are composed of individuals that selection has yet to act upon.

Both age-classes exhibited increases in baseline glucocorticoids following the food restriction, but the effect of the food restriction was much stronger among juveniles. Juveniles had significantly higher food intake than adults, indicating that receiving 25% less of their daily food intake resulted in a loss of relatively more food for juveniles. Additionally, though the proportional change in body condition did not differ between the age classes, juveniles had significantly lower body condition, which reflects lower energy reserves, than adults at each measurement. As energy reserves were relatively more depleted in juveniles, the rise in corticosterone may have been associated with a mobilization of energy derived from protein and lipid catabolism, processes mediated by corticosterone interacting with low affinity type II receptors (Sapolsky 2000; Romero and Wikelski 2001; Landys et al. 2004; Romero 2004). This idea is supported by the negative relationship between body condition and circulating corticosterone found in fasting white-crowned sparrows (*Zonotrichia leucophrys*; Krause et al. 2017), starving Galápagos marine iguanas (*Amblyrhynchus cristatus*; Romero and Wikelski 2001) and here, among juvenile birds. Further analysis of the current data revealed higher post-food restriction baseline corticosterone was associated with lower average body condition and greater declines in body condition after food restriction, highlighting the relevance of consistent individual differences in available energy reserves and acute changes in energy reserves to the dynamics of circulating corticosterone. Interestingly, the relationship was absent before the food restriction, demonstrating how challenging environmental conditions can reveal the consequences of differences in condition to circulating corticosterone (Romero and Wikelski 2010; Benowitz-Fredericks et al. 2022).

In addition to the relationships between food availability, age, and condition, we also found that, following the food restriction, lower baseline corticosterone concentrations occurred in juvenile birds paired with adult social partners with longer telomeres. This indirect relationship may arise because the telomere lengths of adults correlate with either or both their behavioral and physiological response to the food restriction. Indeed, the telomere lengths of adults were significantly correlated with their stress-induced corticosterone concentrations, independent of food availability. Work in other systems has also found relationships between behavior and telomeric traits, including risk taking behavior, foraging behavior, aggressive, social behavior, and migratory behavior (Young et al. 2015; Adriaenssens et al. 2016; Andrews et al. 2018; Vernasco et al. 2021; Vernasco and Watts 2022). Conspecific social cues are also known to be highly relevant for Red Crossbills as they have been found to influence foraging behaviors, modulate the response of the HPA axis to food restriction, and promote adaptive responses to acute declines in food availability (Smith et al. 1999; Cornelius et al. 2010, 2018; Cornelius 2022). The current study builds upon previous works demonstrating the relevance of social cues in Red Crossbills by identifying that baseline glucocorticoids are sensitive to the state of conspecifics, potentially due to adult telomere lengths being associated with differences in activity levels or vocal behavior, for example. By modulating their glucocorticoids based on the state of more experienced social partners, juvenile Red Crossbills may be primed to make more efficient, informed, or adaptive foraging and migratory decisions (Smith et al. 1999; Cornelius et al. 2010, 2018). The lack of a relationship between adult baseline corticosterone and juvenile telomere lengths or body condition may be related to the fact that social cues from inexperienced juveniles are not informative to adults. Indeed, similar age-related patterns of information transfer have been observed during migratory decision making in cranes (*Grus americana*, Abrahms et al. 2021) and in resident killer whales (*Orcinus orca*) experiencing periods of food scarcity (Brent et al. 2015).

The patterns observed in experimental birds differed from those observed in control birds that experienced an identical procedure with the exception that they were last sampled on the day when experimental birds began the food restriction. Specifically, between the two sampling time points, control birds exhibited no significant changes in body condition, a decrease in baseline corticosterone, and no changes in stress-induced corticosterone. Viewed together, the patterns in control birds suggest that the changes in glucocorticoid physiology observed in experimental birds following food restriction are not explained by other aspects of the study protocol. We also observed age-specific differences in baseline corticosterone prior to the food restriction among experimental birds. Adults exhibited significantly higher baseline corticosterone than juveniles 3 days after being moved into an acoustic chamber, a pattern that may reflect age-specific responses to captivity. Juveniles, but not adults, also exhibited significant declines in body condition after being moved into acoustic chambers. Glucocorticoids can either increase or decrease, depending on the species, in response to chronic stressors such as captivity (Dickens and Romero 2013; Romero and Gormally 2019). Interpreting how each age class is responding to captivity is therefore dependent on previous understanding of how chronic stress influences glucocorticoid physiology and the extent to which such effects are age-specific in a given species. As such an understanding is lacking for Red Crossbills, our ability to interpret the age-specific differences in baseline corticosterone prior to the food restriction and the decline in baseline corticosterone in control individuals is limited.

We studied the glucocorticoid responses to an ecologically relevant challenge in a gregarious songbird to understand how extrinsic processes, including food availability and differences in social partners, and intrinsic factors modulate an important endocrine mediator of organismal responses to environmental challenges. Our results demonstrate how the modulation of glucocorticoid concentrations in response to environmental challenges is independent of sex, but dependent on intrinsic differences, including age and condition, as indicated by energy reserves and a proposed biomarker of wear and tear. We also identify how intrinsic differences in social partners can influence glucocorticoid responses to environmental challenges in a nomadic songbird that is known to rely on social cues. Viewed together, we provide a multifaceted understanding of how individual differences in condition and the condition of social partners are associated with glucocorticoid responses to acute and prolonged environmental challenges.

## Supplementary Material

obaf047_Supplemental_File

## Data Availability

All materials necessary to reproduce the results in this study, including data and associated R code, are available at https://doi.org/10.7273/000007814.
