# Supplementary material for: State-Dependent and Social Modulation of Circulating Glucocorticoids in a Nomadic Songbird, the Red Crossbill (Loxia Curvirostra)
Source: Integr Org Biol. 2025 Dec 9;8(1):obaf047. doi: 10.1093/iob/obaf047 (PMC12865310; doi:10.1093/iob/obaf047)
Supplement: obaf047_Supplemental_File [file obaf047_supplemental_file.docx]

**SUPPLEMENTARY MATERIAL**

**Title** State-dependent and social modulation of circulating glucocorticoids in a nomadic songbird, the Red Crossbill (*Loxia curvirostra*)

**Keywords** glucocorticoids, telomeres, body condition, age, nomad, food restriction

**Table of Contents**

**1. Animal Capture and Housing ...................................................................................1**

**2. Relative Telomere Length Measurement .................................................................2**

**3. Testing for Age- and Sex-specific Food Intake and Relative Body Mass Loss**....**4**

**4. Supplementary Figure 1 ............................................................................................6**

**5. Supplementary Figure 2 ............................................................................................8**

**6. Supplementary Figure 3 ............................................................................................9**

**7. Supplementary Figure 4 ..........................................................................................10**

**8. Supplementary Figure 5 ..........................................................................................11**

**9. Supplementary Figure 6 ..........................................................................................12**

**10. Supplementary Table 1 .........................................................................................13**

**11. Supplementary Table 2 .........................................................................................14**

**12. Supplementary Table 3 .........................................................................................16**

**13. Supplementary Table 4 .........................................................................................17**

**14. Supplementary Table 5 .........................................................................................18**

**15. Supplementary Table 6 .........................................................................................19**

**16. Supplementary Table 7 .........................................................................................20**

**17. Supplementary Table 8 .........................................................................................21**

**1. Animal Capture and Housing**

In September and October 2021, 55 wild type 3 Red Crossbills were captured using mist nets at multiple sites within the Mt. Hood and Gifford Pinchot National Forests of Oregon and Washington, respectively. Birds were aged as either juveniles (i.e., < 1 year old) or adult (i.e., 1+ years old) using plumage and morphology as described in [1]. Based on bill size, behavior, and plumage characteristics, juveniles were most likely born between July and September (T. Hahn, personal communication). Birds were sexed using plumage characteristics [1] and juvenile Red Crossbills were genetically sexed by the [*removed for peer-review]* using DNA from blood samples. After capture, birds were transported to [*removed for peer review]* and housed in one of two outdoor aviaries (n = 27 and 29 individuals per aviary) and provided a diet of whole sunflower seeds (i.e., with shells), sunflower hearts, and Roudybush Small Bird Maintenance Diet (Woodland, CA; hereafter Roudybush). All birds had *ad libitum* access to water and grit prior to and during all experimental procedures. Conifer branches and conifer cones were provided in outdoor aviaries for environmental enrichment. On December 1^st^, 2021, birds were moved indoors, housed in individual cages ﻿(l x w x h = 34 x 38.5 x 43 cm), and maintained on a photoperiod mimicking natural changes in day lengths at [*removed for peer-review*], the same latitude as [*removed for peer review*]. All birds had *ad libitum* access to water and grit prior to and during experimental procedures. While indoors, birds were provided with a diet of Roudybush and sunflower hearts *ad libitum* in covered food cups. On December 29th, 2021, cages housing birds were rearranged such that the cages housing individuals that would subsequently be paired with during the experimental procedure were adjacent. Birds were collected under scientific permits from the US Fish and Wildlife Service, Washington Department of Fish and Wildlife, and Oregon Department of Fish and Wildlife. All procedures were approved by the [*removed for peer review*] Institutional Animal Use and Care Committee.

**2. Relative Telomere Length Measurement**

DNA was extracted from red blood cells stored in 100% ethanol using a Gentra Puregene Blood Kit (Qiagen) and a modified extraction protocol. DNA purity and concentration were assessed using a NanoDrop ND-1000 (mean DNA concentration ± SD = 226.1 ± 139.9, range of DNA concentrations = 26.1-823.9 ng/mL, mean 260/280 ratio ± SD = 1.85 ± 0.03, mean 260/230 ratio ± SD = 1.92 ± 0.29). Relative telomere lengths were then quantified using real-time quantitative PCR (qPCR) following the methodologies described in Criscuolo et al. (2009), Eastwood et al. (2018), and Vernasco et al. (2021). Relative telomere lengths are quantified using this approach by comparing amplification kinetics of telomere repeats to those of a single-copy gene, in this case glyceraldehyde-3-phoshate dehydrogenase (GAPDH). Samples were analyzed using a CFX Duet Real-Time PCR System (Bio-Rad) using 96 well plates and a total reaction volume of 15uL. Reactions included 7.5 uL of SsoAdvanced™ Universal SYBR® Green Supermix, 3.6 uL of DNase-free water, 0.15 uL of forward and reverse 100nM telomere or single-copy control gene primers (Sigma-Aldrich), and 3.6 uL of template DNA (2.25 nanograms of DNA per reaction). The amount DNA per reaction was chosen by analyzing different dilutions of the ‘golden’ sample (i.e., a mix of DNA from 5 Red Crossbills not included in the study) and selecting the concentration with optimal well-specific efficiencies [5]. Telomere and GAPDH primers were analyzed on separate plates. Telomere primer sequences were Tel1b 5’ - CGG TTT GTT TGG GTT TGG GTT TGG GTT TGG GTT TGG GTT - 3’ and Tel2b 5’ - GGC TTG CCT TAC CCT TAC CCT TAC CCT TAC CCT TAC CCT - 3’ [4]. The GAPDH primers were developed from the GAPDH sequence of a closely related species (*Spinus pinus*, NCBI KT358792) using the primer design tool in Geneious v10.2.3 (Forward: 5’ - CTG GCA TTG CAC TGA ACG AC - 3’, Reverse: 5’ - GTA GCC AAC AGG ACA CCC AA - 3’; Kearse et al. 2012). Melt curve analyses identified a single peak in all qPCR products. Reaction conditions for telomere primers were as follows: 95°C for 10 min, followed by 40 cycles of 1 minute at 95°C, and 1 minute at 58°C. GAPDH reaction conditions were identical except for the annealing and extension temperature being set to 60°C. Each plate contained all samples belonging to a given individual, a golden sample, and a randomly selected subset of five samples belonging to five different individuals. Two no template controls were also included in each plate and all samples, standards, and controls were run in duplicate.

Cycle quantification (Cq) values and individual well qPCR efficiencies for samples were calculated using *LINREGPCR* version 11 [7]. Samples were excluded if Cq values of duplicates differed by more than > 0.5 [2], though no samples required exclusion using this criterion (mean Cq_telomere_ ± SD = 9.60 ± 0.37, mean Cq_GAPDH_  ± SD = 24.41 ± 0.33, mean efficiency_telomere_ ± SD = 2.03 ± 0.04, mean efficiency_GAPDH_ = 1.91 ± 0.02). Relative telomere lengths (hereafter, telomere length) were calculated following equation one in [8]. Specifically, individual well efficiencies were raised to the power of the ‘golden’ sample Cq value minus the well Cq value. Values from the sample replicates were then averaged and relative telomere lengths were calculated by dividing the average telomere value by the average GAPDH value. Following [9], technical repeatability estimates and their confidence intervals (CIs) were calculated using the duplicate measurements of relative telomere length values and inter-plate repeatability using samples measured on more than one plate with the rptR package within Program R [10]. Technical repeatability was estimated to be 0.94 (95% CIs [0.92, 0.95], p < 0.001) and inter-plate repeatability was estimated to be 0.88 (95% CIs [0.47, 0.96], p < 0.001).

**3. Testing for Age- and Sex-specific Food Intake and Relative Body Mass Loss**

Any observed age- or sex-specific effects of the food restriction may be attributed to age or sex differences in food intake and may also be due to the food restriction being relatively more severe for a particular group. For instance, if one age or sex class exhibits higher food intake, then a 25% reduction in food intake would be a greater amount of food not being provided for that group relative to other groups. Examining the relative decrease in mass loss would also shed insight into whether a particular age or sex group was more severely affected by the food restriction. To test these possibilities, we examined if either food intake or relative body mass loss differed between the age and sex classes using linear models. The response variable for the two models was either average food intake measured before the food restriction (see above for more details) or relative body mass loss. Predictor variables included age, sex, and an interaction between the two. Interaction terms were removed if they were not significant. Relative changes in body mass were calculated by taking the absolute value of post-food intake body mass minus pre-food restriction body mass and dividing the difference by pre-food restriction body mass. Estimated marginal means were calculated from the final model for each age group using the *emmeans* function [11]. Model fit was evaluated using the *check_model* function in the performance package [12]. All models were found to adequately fit the data.

The model examining if food intake differed between age- or sex-classes revealed the interaction term to not be significant (p = 0.35). After removing the interaction term, the simplified model revealed food intake to differ by age classes (β_age_ = 0.21, p = 0.03), but not sex (β_sex_ = 0.09, p = 0.34; Supplementary Figure 6A). The interaction term in the model examining if relative body mass loss significantly differed between age- or sex-classes was also found to be not significant (p = 0.94). The simplified model found that relative body mass loss did not differ by age (β_age_ = -0.03, p = 0.12) or sex (β_sex_ = 0.01, p = 0.65; Supplementary Figure 6B). These results demonstrate that while the food restriction resulted in a greater decrease in food provisions for hatch year individuals, the relative decline in body mass did not significantly differ between the age classes.

**Supplementary Figure 1.** Comparison of changes in body condition and circulating corticosterone in adult (top row) and juvenile (bottom row) Red Crossbills revealed no significant differences in body condition or corticosterone between cages composed of different sex ratios. Colors indicate the cage sex ratio and large circles denote estimated marginal means. Error bars display 95% confidence intervals and thin line show individual data. Data were analyzed using linear mixed models that included an interaction between sampling context (i.e., pre- or post-food restriction) and cage sex ratio. The model examining corticosterone also included an interaction with sample type (i.e., baseline or stress-induced corticosterone). All models included a random effect of individual ID.

**
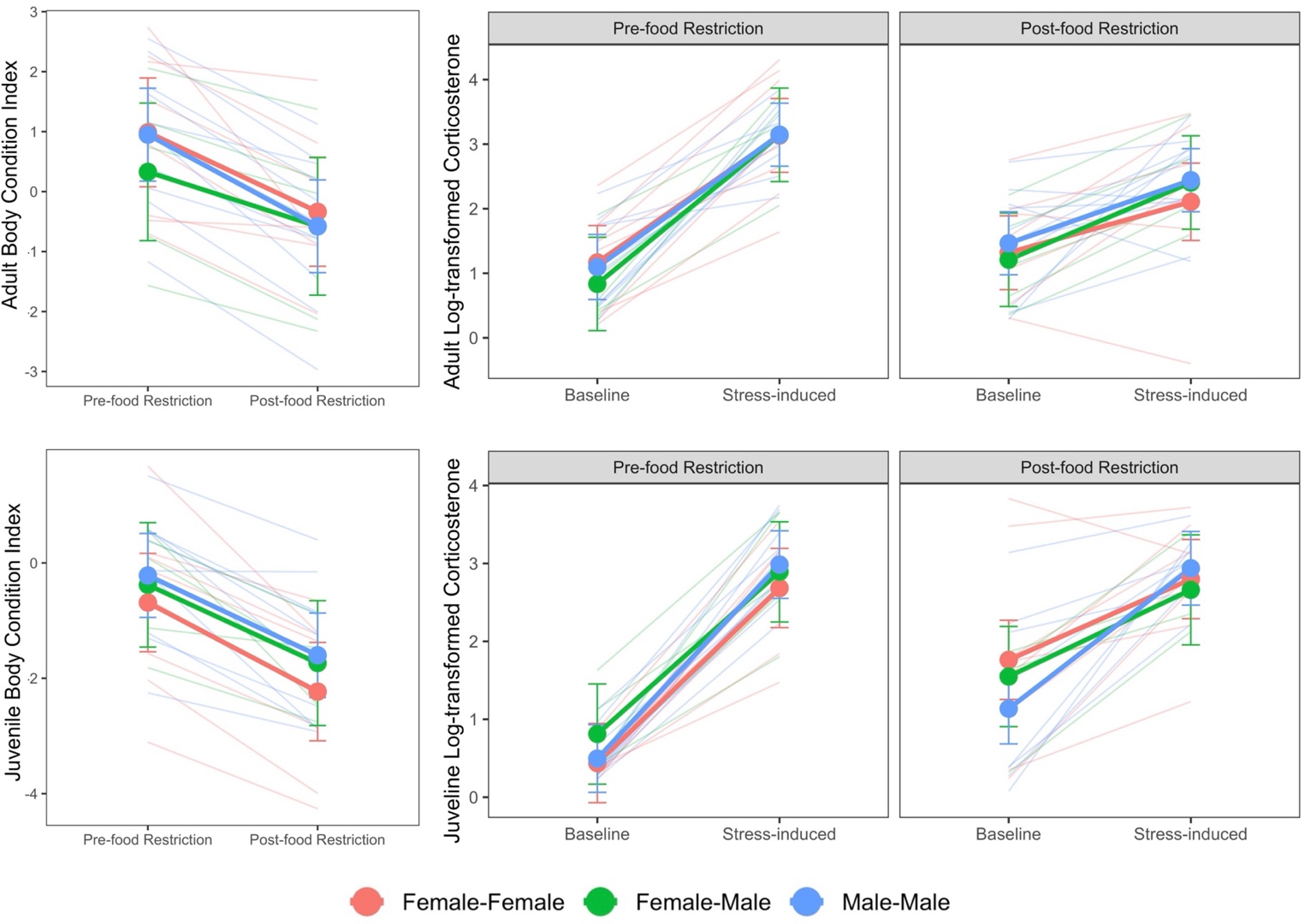
**

**Supplementary Figure 2.** Distribution of and correlations between variables used in statistical analyses. Graphs along the diagonal display distributions of variables. The lower half of the figure displays correlations between variables and the upper half of the figure displays the values of correlation coefficients between predictor variables. Figures were generated using the *ggpairs()* function within the *GGally* v2.1.2 package of Program R [13].

**Supplementary Figure 2A**

**
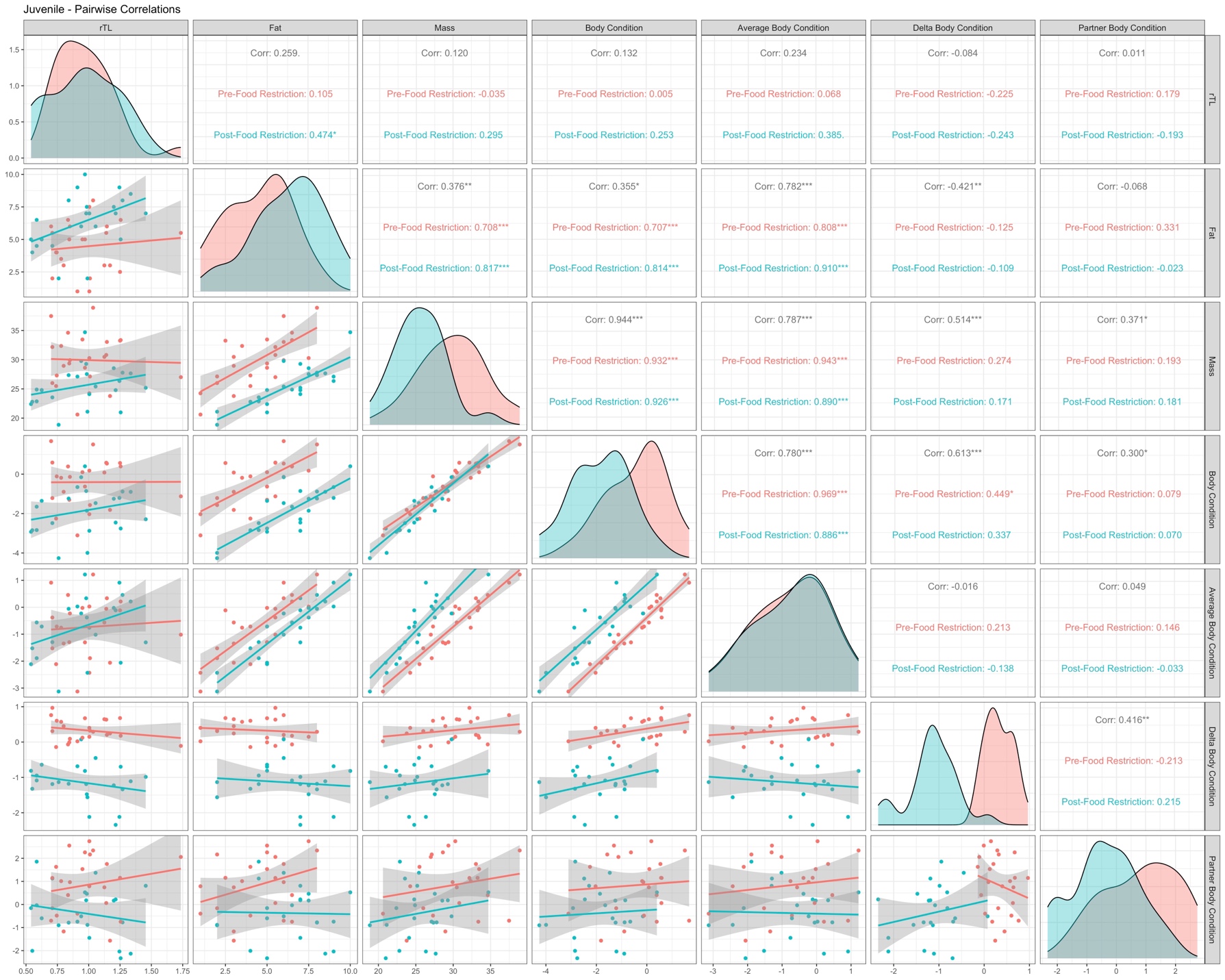
**

**Supplementary Figure 2B**

**
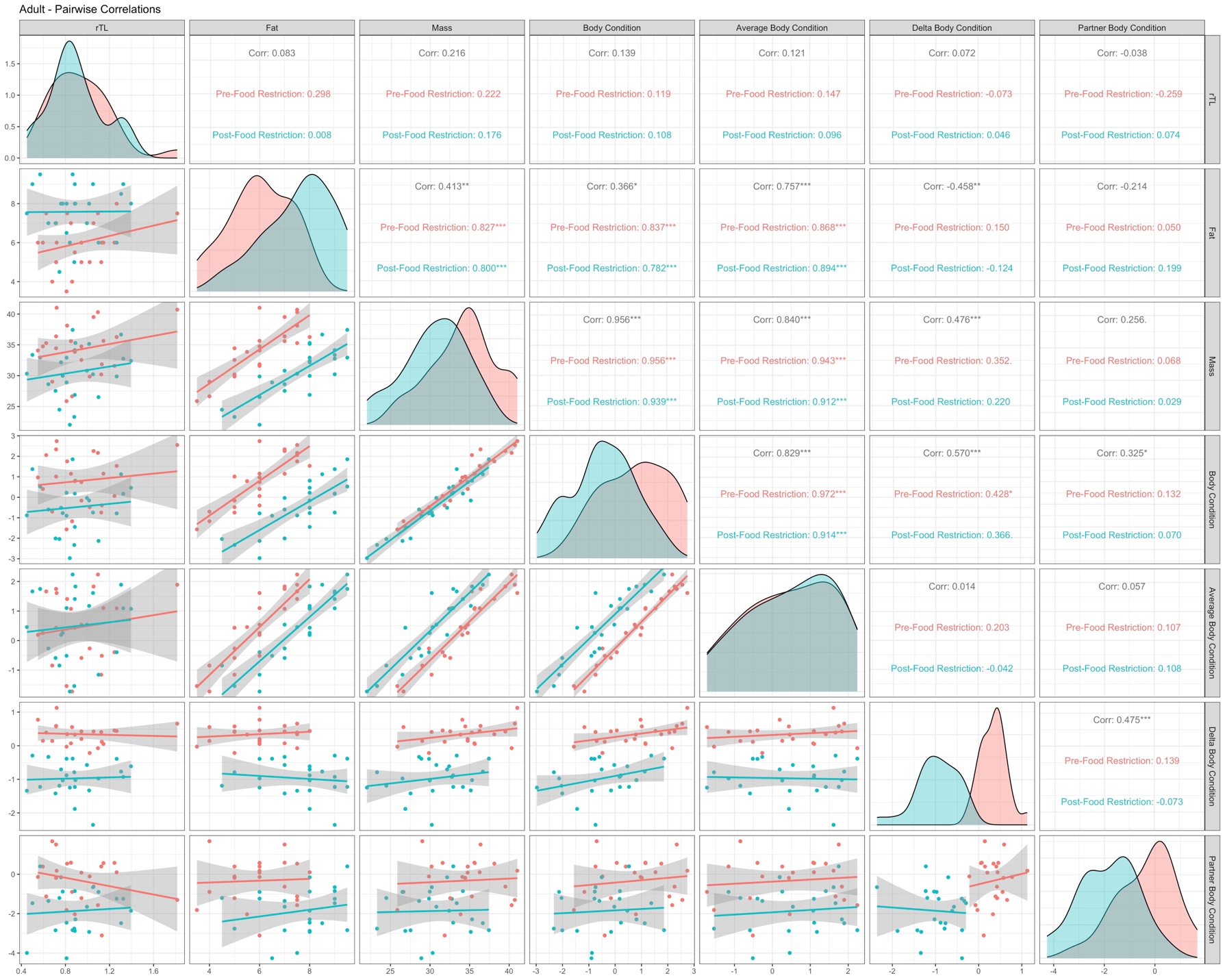
**

**Supplementary Figure 3.** Body condition did not significantly change in control birds over the course of the experiment. Black points and error bars represent estimated marginal means and 95% confidence intervals, respectively, generated from the model summarized in Supplementary Table 1A. Thin lines display connect individual values and color denotes age class.


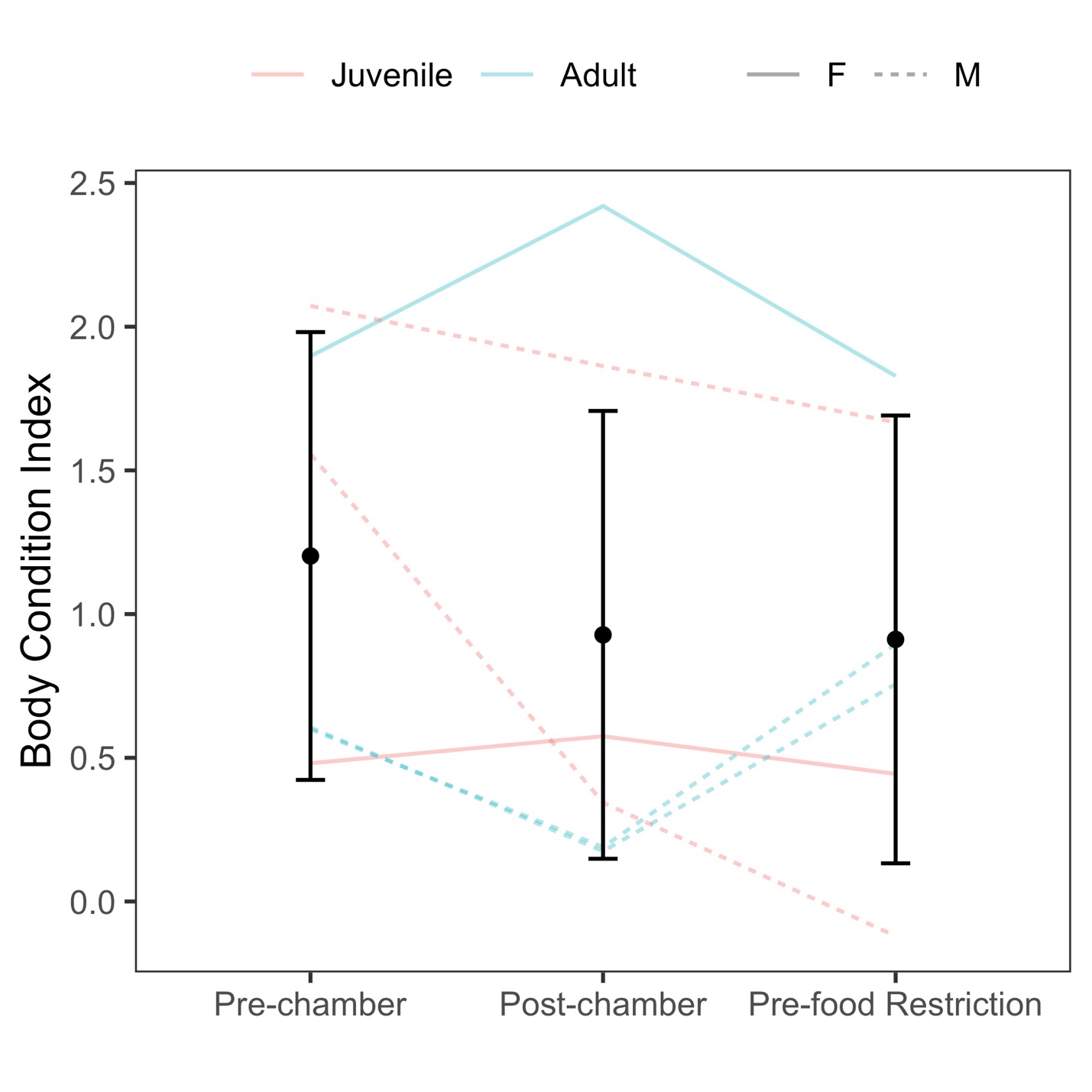


**Supplementary Figure 4.** Relative to values measured from samples collected after 3 days in isolation chambers, baseline values of circulating corticosterone significantly declined in control birds after 10 days in isolation chambers. Stress-induced values did not differ between the two sampling time points. Black points and error bars display estimated marginal means and 95% confidence intervals, respectively, generated from the model summarized in Supplementary Table 1C. Thin lines display connect individual values and color denotes age class.


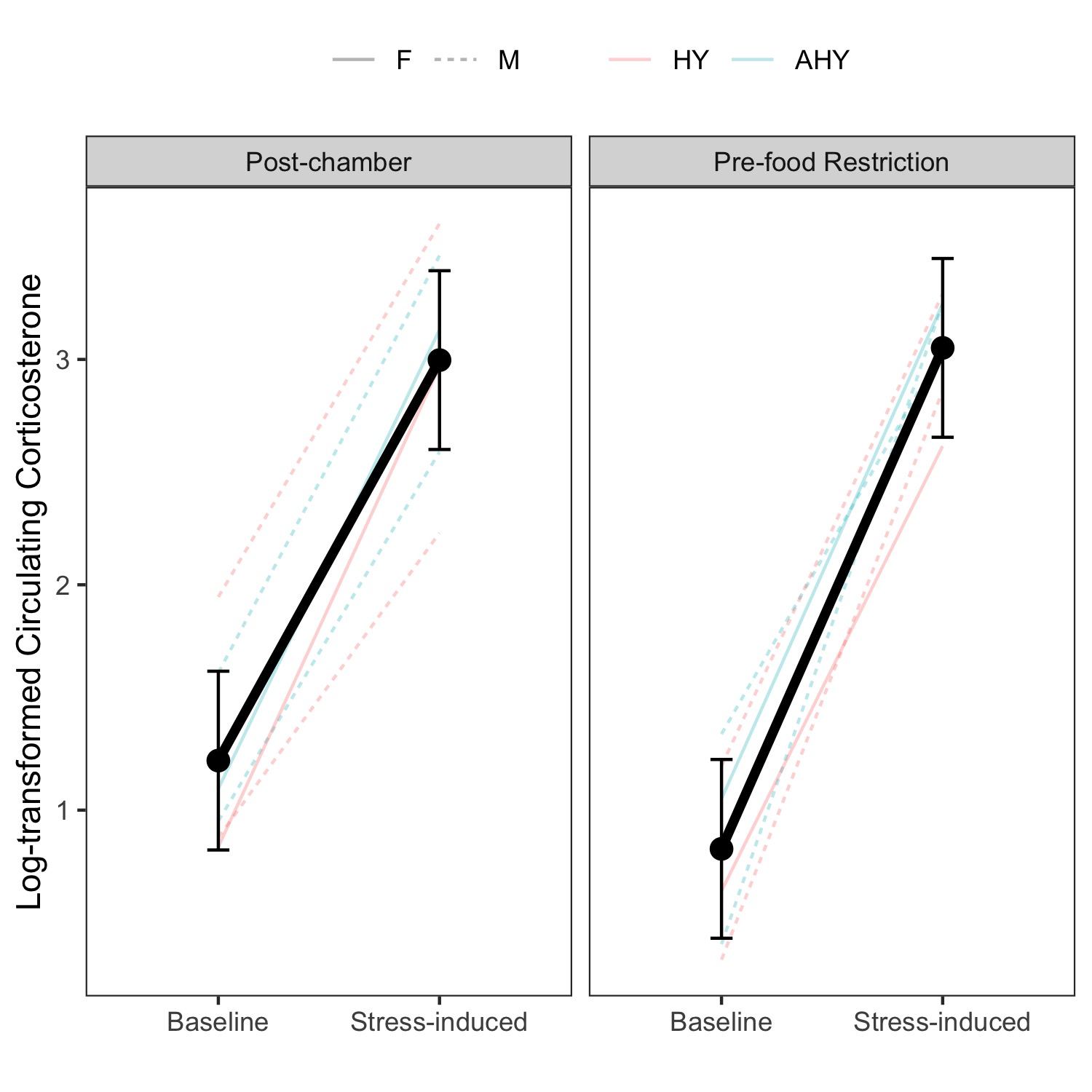


**Supplementary Figure 5.** Post-food restriction, log-transformed juvenile corticosterone values were negatively related to both between-individual differences in body condition (top row) and within-individual changes in juvenile body condition (bottom row). Pre-food restriction there was no relationship with either predictor variable. Points display individual values and lines display the line of best fit generated from the model summarized in Supplementary Table 5A. Inset graphs display slope estimates and their respective 95% confidence intervals.

**
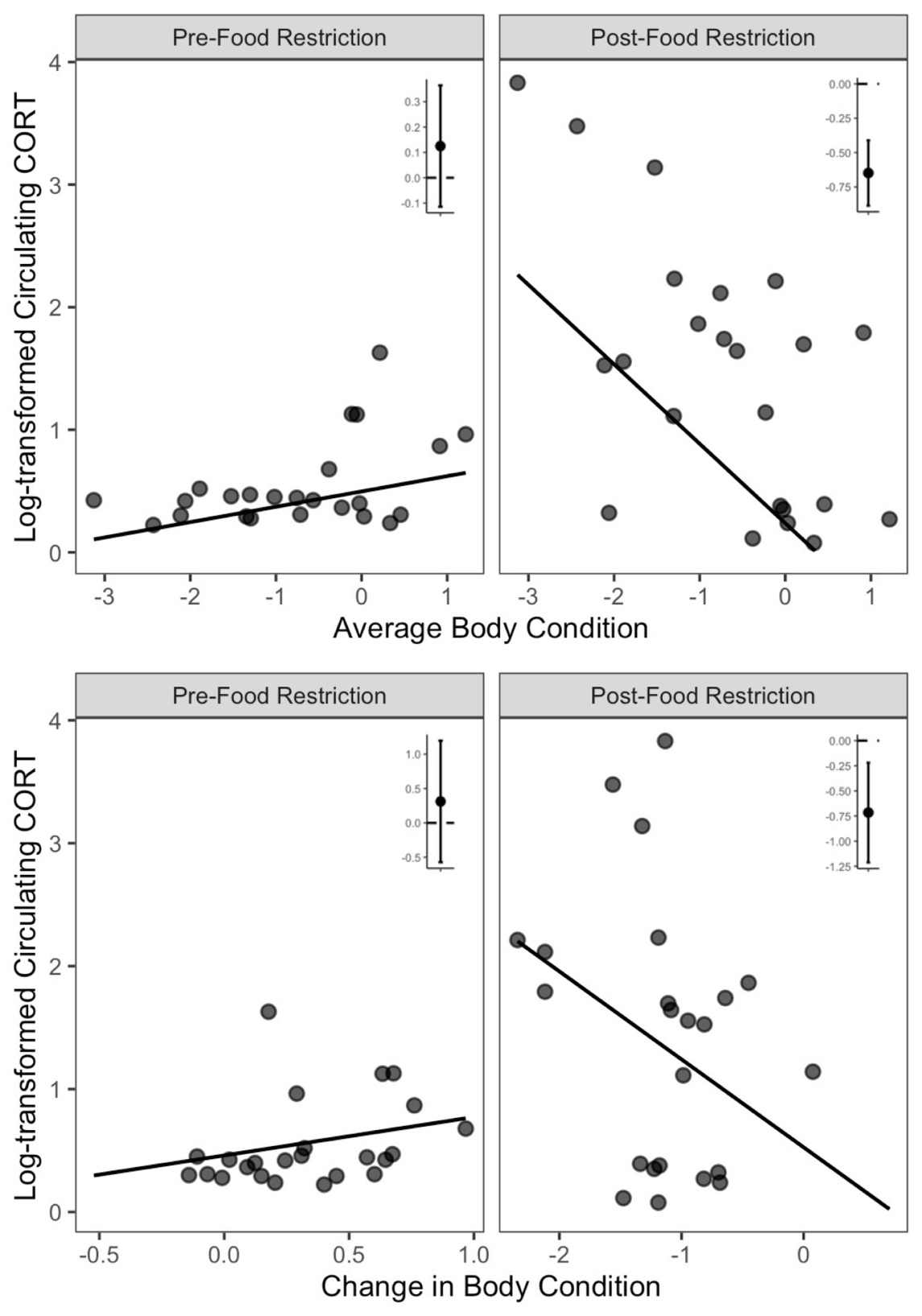
**

**Supplementary Figure 6.** Average daily food intake significantly differed with age, but not sex, before the food restriction (A). Relative body mass loss did not significantly differ with either age or sex (B). Large points reflect estimated marginal means calculated from linear mixed models that include additive effects of age and sex and error bars denote 95% confidence intervals of estimated marginal means. Smaller points show raw data. Color and shape refer to age and sex, respectively. See Section 3 of the Supplementary Text for more details.

**
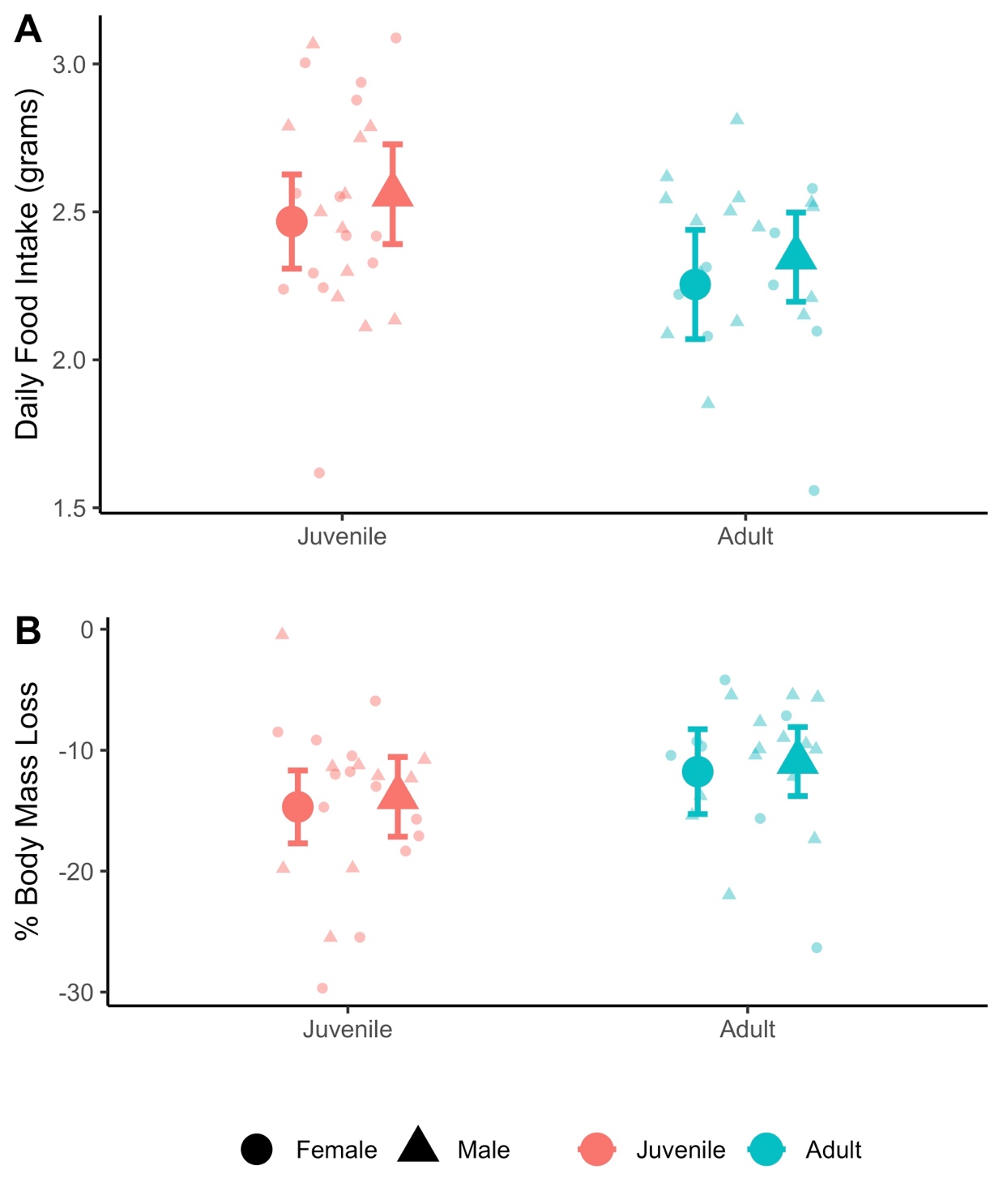
**

**Supplementary Table 1.** Repeatability estimates of predictor variables and glucocorticoid measurements in experimental birds (n = 48 individuals). The body condition index was generated from measurements collected on Day -10, Day -7, and on Day 3. Relative telomere lengths, baseline glucocorticoids, and stress-induced glucocorticoids measurements include samples collected on Day -7 and Day 3. The repeatability of glucocorticoid measurements was calculated after controlling for variation explained by age and sampling context (i.e., pre- or post-food restriction). Control birds (n = 6 individuals) were excluded from all repeatability calculations.

| **Variable** | **Repeatability** | **95% Confidence Intervals** |
| --- | --- | --- |
| Food Intake | 0.56 | 0.41, 0.66 |
| Body Condition Index | 0.55 | 0.39, 0.67 |
| Relative Telomere Length | 0.42 | 0.16, 0.64 |
| Glucocorticoids - Baseline | 0.09 | 0, 0.34 |
| Glucocorticoids - Stress-induced | 0.49 | 0.31, 0.67 |

**Supplementary Table 2.** Summary tables showing results from models examining changes in body condition and circulating corticosterone in control birds.

**Table 2A – Body Condition Model Summary**

| Variable | Estimate | Std. Error | df | t-value | p-value |
| --- | --- | --- | --- | --- | --- |
| Intercept | 1.20 | 0.33 | 7.44 | 3.61 | 0.01 |
| Context: Post-chamber | -0.27 | 0.25 | 10.00 | -1.09 | 0.30 |
| Context: Pre-food Restriction | -0.29 | 0.25 | 10.00 | -1.15 | 0.28 |

**Table 2B – Body Condition Contrasts**

| Contrast | Estimate | SE | df | t-ratio | p-value |
| --- | --- | --- | --- | --- | --- |
| Pre-chamber - Post-chamber | 0.27 | 0.25 | 10 | 1.09 | 0.54 |
| Pre-chamber - Pre-food Restriction | 0.29 | 0.25 | 10 | 1.15 | 0.51 |
| Post-chamber - Pre-food Restriction | 0.02 | 0.25 | 10 | 0.06 | 1 |

**Table 2C – Corticosterone Model Summary**

| Variable | Estimate | Std. Error | df | t-value | p-value |
| --- | --- | --- | --- | --- | --- |
| Intercept | 1.22 | 0.17 | 8.65 | 7.00 | 0.0001 |
| Stress-induced | 1.78 | 0.14 | 15.00 | 12.39 | < 0.0001 |
| Pre-food restriction | -0.39 | 0.14 | 15.00 | -2.73 | 0.02 |
| Stress-induced * Pre-food Restriction | 0.45 | 0.20 | 15.00 | 2.20 | 0.04 |

**Table 2D – Corticosterone Pairwise Comparison**

| Contrast | Estimate | SE | df | t-ratio | p-value |
| --- | --- | --- | --- | --- | --- |
| Baseline: Post-chamber - Pre-food Restriction | 0.39 | 0.14 | 15.00 | 2.73 | 0.02 |
| Stress-induced: Post-chamber - Pre-food Restriction | -0.05 | 0.14 | 15.00 | -0.38 | 0.71 |

**Supplementary Table 3.** (A) Summary table of model testing for age or sex differences in relative telomere lengths among experimental birds at each of the two blood sampling time points. (B) Summary table of models testing for age and sex differences in body condition at each of the three sampling time points. (C) Results from pairwise comparisons from the analysis of changes in body condition amongst experimental birds.

**Table 3A – Relative Telomere Length Model Summary**

| Variable | Estimate | Std. Error | df | t-value | p-value |
| --- | --- | --- | --- | --- | --- |
| Intercept | 0.94 | 0.09 | 40.10 | 10.64 | < 0.0001 |
| Post-food Restriction | -0.13 | 0.09 | 49.04 | -1.41 | 0.17 |
| Age: Juvenile | 0.15 | 0.10 | 86.34 | 1.39 | 0.17 |
| Sex: Male | 0.01 | 0.10 | 87.15 | 0.06 | 0.95 |
| Context * Age: Juvenile | 0.06 | 0.12 | 49.04 | 0.55 | 0.58 |
| Context * Sex: Male | 0.10 | 0.11 | 49.45 | 0.87 | 0.39 |
| Age: Juvenile * Sex: Male | -0.21 | 0.14 | 86.40 | -1.57 | 0.12 |
| Context * Age:Juvenile * Sex:Male | -0.02 | 0.16 | 49.69 | -0.13 | 0.90 |

**Table 3B – Body Condition Model Summary**

| Variable | Estimate | Std. Error | df | t-value | p-value |
| --- | --- | --- | --- | --- | --- |
| Intercept | -0.14 | 0.34 | 57.12 | -0.41 | 0.68 |
| Pre-food Restriction | -0.43 | 0.21 | 88.00 | -2.07 | 0.04 |
| Post-food Restriction | -1.95 | 0.21 | 88.00 | -9.32 | < 0.0001 |
| Age: Adult | 1.43 | 0.53 | 57.12 | 2.71 | 0.01 |
| Sex: Male | 0.49 | 0.50 | 57.12 | 0.98 | 0.33 |
| Pre-food Restriction * Age: Adult | 0.00 | 0.33 | 88.00 | -0.01 | 0.99 |
| Post-food Restriction * Age: Adult | 0.17 | 0.33 | 88.00 | 0.51 | 0.61 |
| Pre-food Restriction * Sex: Male | -0.14 | 0.31 | 88.00 | -0.44 | 0.66 |
| Post-food Restriction * Sex: Male | -0.09 | 0.31 | 88.00 | -0.30 | 0.76 |
| Age: Adult * Sex: Male | -0.79 | 0.72 | 57.12 | -1.10 | 0.28 |
| Pre-food Restriction * Age: Adult * Sex: Male | 0.40 | 0.44 | 88.00 | 0.90 | 0.37 |
| Post-food Restriction * Age: Adult * Sex: Male | 0.39 | 0.44 | 88.00 | 0.88 | 0.38 |

**Table 3B – Body Condition Pairwise Comparisons**

| Sex Contrast | Context | Age | Estimate | SE | df | t-ratio | p-value |
| --- | --- | --- | --- | --- | --- | --- | --- |
| F - M | Pre-chamber | Juvenile | -0.49 | 0.5 | 57.12 | -0.98 | 0.33 |
| F - M | Pre-food Restriction | Juvenile | -0.35 | 0.5 | 57.12 | -0.7 | 0.48 |
| F - M | Post-food Restriction | Juvenile | -0.4 | 0.5 | 57.12 | -0.79 | 0.43 |
| F - M | Pre-chamber | Adult | 0.3 | 0.51 | 57.12 | 0.58 | 0.57 |
| F - M | Pre-food Restriction | Adult | 0.04 | 0.51 | 57.12 | 0.07 | 0.94 |
| F - M | Post-food Restriction | Adult | 0 | 0.51 | 57.12 | 0 | 1 |

| Age Contrast | Context | Age | Estimate | SE | df | t-ratio | p-value |
| --- | --- | --- | --- | --- | --- | --- | --- |
| Juvenile - Adult | Pre-chamber | F | -1.43 | 0.53 | 57.12 | -2.71 | 0.01 |
| Juvenile - Adult | Pre-food Restriction | F | -1.43 | 0.53 | 57.12 | -2.7 | 0.01 |
| Juvenile - Adult | Post-food Restriction | F | -1.6 | 0.53 | 57.12 | -3.03 | 0.004 |
| Juvenile - Adult | Pre-chamber | M | -0.65 | 0.48 | 57.12 | -1.34 | 0.19 |
| Juvenile - Adult | Pre-food Restriction | M | -1.04 | 0.48 | 57.12 | -2.15 | 0.04 |
| Juvenile - Adult | Post-food Restriction | M | -1.2 | 0.48 | 57.12 | -2.48 | 0.02 |

| Context Contrast | Context | Age | Estimate | SE | df | t-ratio | p-value |
| --- | --- | --- | --- | --- | --- | --- | --- |
| Pre-chamber - Pre-food Restriction | Juvenile | F | 0.43 | 0.21 | 88 | 2.07 | 0.04 |
| Pre-chamber - Pre-food Restriction | Adult | F | 0.44 | 0.25 | 88 | 1.74 | 0.09 |
| Pre-chamber - Pre-food Restriction | Juvenile | M | 0.57 | 0.23 | 88 | 2.51 | 0.01 |
| Pre-chamber - Pre-food Restriction | Adult | M | 0.18 | 0.19 | 88 | 0.91 | 0.37 |
| Pre-food restriction - Post-food Restriction | Juvenile | F | 1.51 | 0.21 | 88 | 7.25 | < 0.0001 |
| Pre-food restriction - Post-food Restriction | Adult | F | 1.34 | 0.25 | 88 | 5.35 | < 0.0001 |
| Pre-food restriction - Post-food Restriction | Juvenile | M | 1.47 | 0.23 | 88 | 6.47 | < 0.0001 |
| Pre-food restriction - Post-food Restriction | Adult | M | 1.31 | 0.19 | 88 | 6.72 | < 0.0001 |
| Pre-chamber - Post-food Restriction | Juvenile | F | 1.95 | 0.21 | 88 | 9.32 | < 0.0001 |
| Pre-chamber - Post-food Restriction | Adult | F | 1.78 | 0.25 | 88 | 7.09 | < 0.0001 |
| Pre-chamber - Post-food Restriction | Juvenile | M | 2.04 | 0.23 | 88 | 8.98 | < 0.0001 |
| Pre-chamber - Post-food Restriction | Adult | M | 1.48 | 0.19 | 88 | 7.62 | < 0.0001 |

**Supplementary Table 4.** Summary tables of top supported models examining the relationship between circulating corticosterone and predictor variables representing focal individual traits.

**Supplementary Table 4A – Juvenile Baseline Corticosterone**

| Variable | Estimate | Std. Error | df | t-value | p-value |
| --- | --- | --- | --- | --- | --- |
| Intercept | 0.60 | 0.13 | 42.38 | 4.68 | 0.00003 |
| Body Condition | 0.14 | 0.10 | 42.95 | 1.40 | 0.17 |
| Post-food Restriction | -0.38 | 0.26 | 32.76 | -1.48 | 0.15 |
| Body Condition * Post-food Restriction | -0.80 | 0.14 | 25.32 | -5.69 | 0.00001 |

**Supplementary Table 4B – Juvenile Stress-induced Corticosterone**

| Variable | Estimate | Std. Error | df | t-value | p-value |
| --- | --- | --- | --- | --- | --- |
| Intercept | 2.87 | 0.12 | 32.43 | 23.59 | < 0.00001 |
| Post-food Restriction | -0.03 | 0.11 | 21.76 | -0.29 | 0.78 |

**Supplementary Table 4C – Adult Baseline Corticosterone**

| Variable | Estimate | Std. Error | df | t-value | p-value |
| --- | --- | --- | --- | --- | --- |
| Intercept | 1.07 | 0.16 | 40.43 | 6.81 | < 0.00001 |
| Post-food Restriction | 0.30 | 0.18 | 22.18 | 1.68 | 0.11 |

**Supplementary Table 4D – Adult Stress-induced Corticosterone**

| Variable | Estimate | Std. Error | df | t-value | p-value |
| --- | --- | --- | --- | --- | --- |
| Intercept | 2.01 | 0.40 | 42.25 | 5.07 | 0.00001 |
| Relative Telomere Length | 1.15 | 0.39 | 39.75 | 2.98 | 0.005 |
| Post-food Restriction | -0.71 | 0.12 | 20.74 | -5.68 | 0.00001 |

**Supplementary Table 5.** Summaries from models examining the relationship between baseline circulating corticosterone and body condition in juvenile birds. In these models, body condition was decomposed into between-individual (i.e., average body condition) and within-individual effects (i.e., delta-body condition). Supplementary Table 4B is used to examine the extent to which any between- and within-individual effects are statistically different. See van de Pol and Wright (2009) for a full description of this modeling approach.

**Supplementary Table 5A**

| Variables | Estimate | Std. Error | df | t-value | p-value |
| --- | --- | --- | --- | --- | --- |
| Intercept | 0.53 | 0.22 | 40.98 | 2.42 | 0.02 |
| Post-food Restriction | -0.37 | 0.37 | 28.69 | -1.00 | 0.33 |
| Average Body Condition | 0.13 | 0.12 | 40.34 | 1.06 | 0.30 |
| Delta-Body Condition | 0.31 | 0.42 | 40.72 | 0.74 | 0.46 |
| Post-food Restriction * Average Body Condition | -0.77 | 0.15 | 21.03 | -5.01 | 0.0001 |
| Post-food Restriction * Delta-Body Condition | -1.03 | 0.50 | 37.69 | -2.06 | 0.05 |

**Supplementary Table 5B**

| Variables | Estimate | Std. Error | df | t-value | p-value |
| --- | --- | --- | --- | --- | --- |
| Intercept | 0.53 | 0.22 | 40.98 | 2.42 | 0.02 |
| Post-food Restriction | -0.37 | 0.37 | 28.69 | -1.00 | 0.33 |
| Average Body Condition | -0.19 | 0.46 | 40.80 | -0.41 | 0.69 |
| Body Condition | 0.31 | 0.42 | 40.72 | 0.74 | 0.46 |
| Post-food Restriction * Average Body Condition | 0.25 | 0.53 | 39.80 | 0.48 | 0.64 |
| Post-food Restriction * Body Condition | -1.03 | 0.50 | 37.69 | -2.06 | 0.05 |

**Supplementary Table 6.** Model selection tables quantifying support for variables measured from an individual’s social partner.

**Supplementary Table 6A – Baseline Juvenile**

| Model | Params | ΔAICc | weight |
| --- | --- | --- | --- |
| Context * Partner rTL | 6 | 0.00 | 0.74 |
| Context + Partner rTL | 5 | 3.42 | 0.13 |
| Context | 4 | 3.70 | 0.12 |
| Context + Partner Body Condition | 5 | 7.97 | 0.01 |
| Context * Partner Body Condition | 6 | 11.71 | 0.00 |

**Supplementary Table 6B – Stress-induced Juvenile**

| Model | Params | ΔAICc | weight |
| --- | --- | --- | --- |
| Context | 4 | 0.00 | 0.55 |
| Context + Partner rTL | 5 | 1.21 | 0.30 |
| Context * Partner rTL | 6 | 3.25 | 0.11 |
| Context + Partner Body Condition | 5 | 5.69 | 0.03 |
| Context * Partner Body Condition | 6 | 11.07 | 0.00 |

**Supplementary Table 6C – Baseline Adult**

| Model | Params | ΔAICc | weight |
| --- | --- | --- | --- |
| Context | 4 | 0.00 | 0.55 |
| Context + Partner rTL | 5 | 1.89 | 0.21 |
| Context * Partner rTL | 6 | 2.39 | 0.17 |
| Context + Partner Body Condition | 5 | 4.77 | 0.05 |
| Context * Partner Body Condition | 6 | 6.866 | 0.02 |

**Supplementary Table 6D – Stress-induced Adult**

| Model | Params | ΔAICc | weight |
| --- | --- | --- | --- |
| Context | 4 | 0.00 | 0.45 |
| Context + Partner Body Condition | 5 | 1.62 | 0.20 |
| Context * Partner rTL | 6 | 2.25 | 0.15 |
| Context + Partner rTL | 5 | 2.50 | 0.13 |
| Context * Partner Body Condition | 6 | 3.72 | 0.07 |

**Supplementary Table 7.** Summary of top-supported model examining the relationship between social partner relative telomere lengths and baseline circulating corticosterone measured from juveniles.

| Variable | Estimate | Std. Error | df | t-value | p-value |
| --- | --- | --- | --- | --- | --- |
| Intercept | 0.38 | 0.57 | 42.00 | 0.67 | 0.51 |
| Post-Food Restriction | 2.62 | 0.82 | 42.00 | 3.20 | 0.003 |
| Partner rTL | 0.18 | 0.58 | 42.00 | 0.32 | 0.75 |
| Post-food Restriction * Partner rTL | -1.91 | 0.86 | 42.00 | -2.23 | 0.03 |

**Supplementary Table 8.** Model selection tables quantifying support for variables representing focal individual (as opposed to partner) traits.

**Supplementary Table 8A – Juvenile Baseline Corticosterone**

| Model | Params | ΔAICc | weight |
| --- | --- | --- | --- |
| Context * Body Condition | 6 | 0.00 | 1.00 |
| Context * rTL | 6 | 17.75 | 0.00 |
| Context + Body Condition | 5 | 18.43 | 0.00 |
| Context | 4 | 19.11 | 0.00 |
| Context + rTL | 5 | 20.15 | 0.00 |

**Supplementary Table 8B – Juvenile Stress-induced Corticosterone**

| Model | Params | ΔAICc | weight |
| --- | --- | --- | --- |
| Context | 4 | 0.00 | 0.62 |
| Context * rTL | 6 | 2.59 | 0.17 |
| Context + rTL | 5 | 2.80 | 0.15 |
| Context + Body Condition | 5 | 4.95 | 0.05 |
| Context * Body Condition | 6 | 7.89 | 0.01 |

**Supplementary Table 8C – Adult Baseline Corticosterone**

| Model | Params | ΔAICc | weight |
| --- | --- | --- | --- |
| Context | 4 | 0.00 | 0.62 |
| Context + rTL | 5 | 2.16 | 0.21 |
| Context * rTL | 6 | 3.48 | 0.11 |
| Context + Body Condition | 5 | 5.26 | 0.04 |
| Context * Body Condition | 6 | 7.24 | 0.02 |

**Supplementary Table 8D – Adult Stress-induced Corticosterone**

| Model | Params | ΔAICc | weight |
| --- | --- | --- | --- |
| Context + rTL | 5 | 0.00 | 0.53 |
| Context * rTL | 6 | 0.41 | 0.43 |
| Context | 4 | 5.58 | 0.03 |
| Context + Body Condition | 5 | 10.38 | 0.00 |
| Context * Body Condition | 6 | 14.10 | 0.00 |

**REFERENCES**

1. Pyle P. 1997 *Identification guide to North American birds: Part I Columbidae to Ploceidae*. Bolinas, CA: Slate Creek Press.

2. Eastwood JR, Mulder E, Verhulst S, Peters A. 2018 Increasing the accuracy and precision of relative telomere length estimates by RT qPCR. *Mol. Ecol. Resour.* **18**, 68–78. (doi:10.1111/1755-0998.12711)

3. Vernasco BJ, Dakin R, Majer AD, Haussmann MF, Brandt Ryder T, Moore IT. 2021 Longitudinal dynamics and behavioural correlates of telomeres in male wire‐tailed manakins. *Funct. Ecol.* **35**, 450–462. (doi:10.1111/1365-2435.13715)

4. Criscuolo F, Bize P, Nasir L, Metcalfe NB, Foote CG, Griffiths K, Gault E a., Monaghan P. 2009 Real-time quantitative PCR assay for measurement of avian telomeres. *J. Avian Biol.* **40**, 342–347. (doi:10.1111/j.1600-048X.2008.04623.x)

5. Morinha F, Magalhães P, Blanco G. 2020 Standard guidelines for the publication of telomere qPCR results in evolutionary ecology. *Mol. Ecol. Resour.* **20**, 635–648. (doi:10.1111/1755-0998.13152)

6. Kearse M *et al.* 2012 Geneious Basic: an integrated and extendable desktop software platform for the organization and analysis of sequence data. *Bioinformatics* **28**, 1647–1649. (doi:10.1093/bioinformatics/bts199)

7. Ruijter JM, Ramakers C, Hoogaars WMH, Karlen Y, Bakker O, van den Hoff MJB, Moorman AFM. 2009 Amplification efficiency: linking baseline and bias in the analysis of quantitative PCR data. *Nucleic Acids Res.* **37**, e45–e45. (doi:10.1093/nar/gkp045)

8. Pfaffl MW. 2001 A new mathematical model for relative quantification in real-time RT-PCR. *Nucleic Acids Res.* **29**, 45e – 45. (doi:10.1093/nar/29.9.e45)

9. Kärkkäinen T *et al.* 2021 Population differences in the length and early‐life dynamics of telomeres among European pied flycatchers. *Mol. Ecol.* , 1–13. (doi:10.1111/mec.16312)

10. Stoffel MA, Nakagawa S, Schielzeth H. 2017 rptR: repeatability estimation and variance decomposition by generalized linear mixed‐effects models. *Methods Ecol. Evol.* **8**, 1639–1644. (doi:10.1111/2041-210X.12797)

11. Lenth R V. 2022 emmeans: Estimated Marginal Means, aka Least-Squares Means.

12. Lüdecke D, Ben-Shachar M, Patil I, Waggoner P, Makowski D. 2021 performance: An R Package for Assessment, Comparison and Testing of Statistical Models. *J. Open Source Softw.* **6**, 3139. (doi:10.21105/joss.03139)

13. Schloerke B, Cook D, Larmarange J, Briatte F, Marbach M, Thoen E, Elberg A, Crowley J. 2021 GGally: Extension to ‘ggplot2’.

14. van de Pol M, Wright J. 2009 A simple method for distinguishing within- versus between-subject effects using mixed models. *Anim. Behav.* **77**, 753–758. (doi:10.1016/j.anbehav.2008.11.006)
